# Long noncoding RNA DLEU2 and ROR1 pathway induces epithelial-to-mesenchymal transition and cancer stem cells in breast cancer

**DOI:** 10.1038/s41420-024-01829-3

**Published:** 2024-01-31

**Authors:** Syed S. Islam, Taher Al-Tweigeri, Layla Al-Harbi, Shafat Ujjahan, Maha Al-Mozaini, Asma Tulbah, Abdelilah Aboussekhra

**Affiliations:** 1 Department of Molecular Oncology, King Faisal Specialist Hospital & Research Centre, Riyadh, Saudi Arabia; 2 School of Medicine, Al-Faisal University, Riyadh, Saudi Arabia; 3 Breast Cancer Unit, Oncology Centre, King Faisal Specialist Hospital & Research Centre, Riyadh, Saudi Arabia; 4 Department of Infection and Immunity, King Faisal Specialist Hospital & Research Centre, Riyadh, Saudi Arabia; 5 Department of Medical Oncology and Radiotherapy, Park View Hospital, Chattagram, Bangladesh; 6 Department of Pathology and Laboratory Medicine, King Faisal Specialist Hospital & Research Centre, Riyadh, Saudi Arabia

**Keywords:** Breast cancer, Predictive markers

## Abstract

Breast cancer (BC) patient who receives chemotherapy for an extended length of time may experience profound repercussions in terms of metastases and clinical outcomes due to the involvement of the epithelial-to-mesenchymal transition (EMT) mechanism and enriched cancer stem cells (CSCs). BC cells that express high levels of lncRNA deleted in lymphocytic leukemia-2 (lncRNA DLEU2) and type I tyrosine kinase-like orphan receptor ROR1 (ROR1) may play roles in the enhanced ability of the activation EMT and CSC induction. Here we find that lncRNA DLEU2 and ROR1 are specifically upregulated in tumor tissues compared to their normal counterparts in TCGA, PubMed GEO datasets, and samples from archived breast cancer tumor tissues. Following chemotherapy, lncRNA DLEU2 and ROR1 were enhanced in BC tumor cells, coupled with the expression of CSCs, EMT-related genes, and BMI1. Mechanistically, ROR1 and lncRNA DLEU2 overexpression led to enhanced tumor cell proliferation, inhibition of apoptosis, cell-cycle dysregulation, chemoresistance, as well as BC cell’s abilities to invade, migrate, develop spheroids. These findings imply that the role of lncRNA DLEU2 and ROR1 in BC therapeutic failure is largely attributed to EMT, which is intricately linked to enriched CSCs. In conclusion, our findings indicate that a lncRNA DLEU2 and ROR1-based regulatory loop governs EMT and CSC self-renewal, implying that targeting this regulatory pathway may improve patients’ responses to chemotherapy and survival.

## Background

Breast cancer (BC) is the most frequently diagnosed cancer and the second leading cause of malignancy-related death among women in both developing and developed countries [1]. The treatment regimens for BC vary depending on the cancer subtype and stage at diagnosis. Although BC patients are treated with surgery, chemotherapy, hormone therapy, and radiation therapy [1, 2] the prognosis has not significantly improved despite recent medical advancement. BC enduring chemotherapies have been reported to enrich greater mesenchymal and stemness features enabling cells to metastasize and tumor relapse [3]. Thus, identifying new target genes and related pathways for disease treatment renders an added advantage to exploring the molecular mechanisms related to BC therapeutic resistance, which will lead to improved personalized therapeutic design.

Long noncoding RNAs’ (lncRNAs) involvement in cancer metastasis is attributed to the regulation of epithelial-to-mesenchymal transition (EMT), which is intricately linked to the enrichment of cancer stem cells (CSCs). Recent research has identified a handful of lncRNAs that are implicated in the Wnt/beta-catenin, hedgehog (HH), Notch, and TGF-β signaling pathways, where they individually or collectively regulate the transcription factors involved in stemness induction and maintenance [4]. Evidence from several preclinical reports regarding lncRNA antagonists exhibited promising outcomes for cancer treatment. For example, inhibition of lncRNAs by modifying antisense oligonucleotides (GapmeRs) has proven efficacy in inhibiting tumor growth and metastasis and sensitizing tumors to chemotherapeutic drugs [5–7].

LncRNA DLEU2 (lncRNA deleted in lymphocytic leukemia-2) has been identified in numerous cancers as a tumor suppressor RNA, suggesting that the lncRNA DLEU2 may be used as a molecular marker for diagnosis and treatment [8]. Furthermore, abnormal lncRNA DLEU2 mutations play a crucial role in tumor progression in pancreatic, lung, and hematopoietic malignancies [9–11]. These findings signify the multifaceted functions that lncRNA DLEU2 plays in a range of malignancies. However, the regulatory roles and functions of lncRNA DLEU2 in EMT, CSCs, and the chemoresistance of BC cells are unknown.

The receptor tyrosine kinase (RTK)-like orphan receptors (RORs) serve as extracellular ligand-binding domains. However, their ligands, cellular outcomes, and interactions with long noncoding RNAs are largely unknown. Mounting evidence has reported that the ROR proteins are highly expressed in leukemia, ovarian, and BC [12, 13]. Among the two ROR proteins (ROR1 and ROR2), prior studies found that BC with high levels of ROR1 is associated with cancer stemness and EMT, rapid disease progression after chemotherapy, and short survival [13]. Additionally, it has been suggested that silencing ROR1 could potentially suppress the genes linked to EMT, impaired cellular migration, invasion, and metastasis [14, 15].

In this study, we examined the expression of lncRNA DLEU2 and ROR1 in human BC patients and compared them with normal breast tissues. In addition, we examined the expression of these genes in BC patients’ tumors who were resistant or sensitive to chemotherapy. Furthermore, we validated our results with PubMed GEO and TCGA datasets. All of these indicates that the lncRNA DLEU2 and ROR1 is elevated in BC tumors, particularly in patients who are resistant to chemotherapy. In addition, we investigated the functional involvement of the lncRNA DLEU2 and ROR1 in inducing EMT, CSC, and chemoresistance using in vitro experiments.

## Results

### LncRNA DLEU2 is upregulated in breast cancer tissues and cell lines

First, to investigate the expression of lncRNA DLEU2, we compared the expression levels of lncRNA DLEU2 in normal and human BC tissues using multiple datasets from the ONCOMINE database. We have chosen a *P*-value threshold parameter of 0.01, a fold change parameter of 1.5, and a gene rank of 15%. Using these criteria, we have found that the expression of lncRNA DLEU2 is significantly higher in tumors from BC patients than with normal tissues across all datasets. LncRNA DLEU2 was highly elevated in invasive ductal breast carcinoma tissues in the Curtis [16], Richardson [17], and the Turashvili datasets [18] with a fold-change of 2.24, 1.82, 1.79, and 2.99, respectively. In contrast, however, the Zhao [19], and Gluck [20] datasets showed comparatively lower expression of lncRNA DLEU2 in BC tissues, indicating that there may be variations in lncRNA DLEU2 expression between various datasets. In the Curtis [16] dataset, lncRNA DLEU2 was highly expressed in invasive lobular breast carcinoma tissues with a fold-change of 1.87. Further details on lncRNA DLEU2 and several additional clinically significant lncRNAs are included in Supplementary Table S1 together with information on their expression and fold-change significance values.

**Fig. 1:**
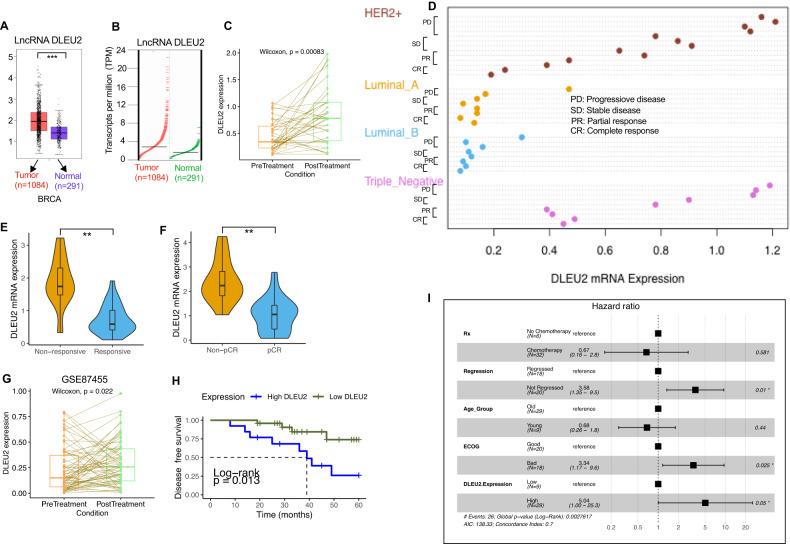
High expression of lncRNA DLEU2 is associated with breast cancer patients’ treatment outcomes. **A** (Boxplot), and **B** (Scatter diagram). The differential expression of lncRNA DLEU2 in breast tumors and normal tissues (The data were retrieved and analyzed from the GEPIA database). **C** LncRNA DLEU2 expression of pre-and-post-treatment breast cancer patients (*n* = 38) treated with four cycles of epirubicin combined with chemotherapy (Wilcoxon test, *P* = 0.00083). **D** The lncRNA DLUE2 mRNA expression in different molecular subtypes and different responses to chemotherapy including complete response (CR), partial response (PR), stable disease (SD), and progressive disease (PD) in breast cancer (*n* = 38) patients. Patients were treated with neoadjuvant chemotherapy as described in the materials and methods section. **E** Relative lncRNA DLEU2 expression levels in response to chemotherapy (**P < 0.01). **F** Violin plot showing the expression levels of lncRNA DLEU2 in breast cancer patients with pCR (pathological complete response) compared with those of non-pCR patient groups (**P < 0.01). **G** LncRNA DLEU2 expression of pre-and-post-treatment breast cancer patients treated with four cycles of epirubicin combined with chemotherapy (Wilcoxon test, *P* = 0.022). GSE84755, Kimbung et al. [3]. **H** The Kaplan–Meier disease-free survival curve of advanced breast cancer patients classified as low- and -high lncRNA-DLEU2 groups based on the median expression level of lncRNA DLEU2. **I** The association between lncRNA DLEU2 expression and the clinical parameters of breast cancer patients by multivariate analysis.

We then examined how the expression of lncRNA DLEU2 differs between tumors and normal samples using the GEPIA (Gene Expression Profiling Interactive Analysis) dataset. Figure 1A, B shows that, in contrast to normal samples, BC tissues had higher levels of lncRNA DLEU2 expression (*P* < 0.001). Likewise, different clinical stages of breast tumors have varying levels of lncRNA DLEU2 expression (Fig. S1A). Using the Cancer Cell Lines Encyclopedia (CCLE) database, we have broadened the scope of our investigation into the differential expression of lncRNA DLEU2 in various types of cancers. We have detected notable differences in the expression of lncRNA DLEU2 between BC tumors and other tumor tissues. Notably, lncRNA DLEU2 was highly expressed in BC tissues (Fig. S1B). Additionally, we explored the expression of lncRNA DLEU2 in a range of BC cell lines using the bioinformatics tools provided by the European Bioinformatics Institute (EMBL-EBL) (https://www.edi.ac.uk/gxa/home). The results revealed that lncRNA DLEU2 is highly expressed in a substantial number of BC cell lines (Fig. S1C). Furthermore, it was determined that a high level of lncRNA DLEU2 was associated with poor overall survival (OS) and disease-free survival (DFS), although without reaching the significance level, as depicted in Fig. S1D, W. We used the LinkedOmics database to compare and determine the significance of lncRNA DLEU2 in patients’ OS to validate the GEPIA results. Surprisingly, there were no discrepancies between GEPIA and LinkedOmics datasets (data not presented).

Following that, the functional role of lncRNA DLEU2 and the genes significantly related to lncRNA DLEU2 alterations were predicted by analyzing the gene ontology (GO) in the Database for Annotation, Visualization, and Integrated Discovery (DAVID). GO analysis provides defined GO terms for genes. At the highest level, GO terms cover biological processes (BP), cellular components (CC), and molecular functions (MF). Due to its suitability for enrichment analysis of gene sets, it has become one of the most popular annotation sources. In our analysis, several significant GO-BP genes were found to be regulated by the lncRNA-DLEU2 alteration in BC (Fig. S1F).

### High expression of lncRNA DLEU2 is associated with BC chemoresistance

We obtained 38 biopsy tissues from patients with invasive ductal breast adenocarcinoma. The expression level of lncRNA DLEU2 was measured in 38 cases of BC tissue samples obtained from patients before and after neoadjuvant chemotherapy (Supplementary Table S2). These patients received three cycles of a combination of doxorubicin, docetaxel, or epirubicin with/or without cyclophosphamide (Supplementary Table S2). The expression of lncRNA DLEU2 was detected in all 38 cases of BC tissue samples by qRT-PCR. We found that lncRNA DLEU2 expression was increased in 23 (60%) of 38 matched samples (this includes ER+ and/or ER- BC samples) (Fig. 1C, *P* = 0.00083). The expression was decreased in 10 (26%) of 38- matched posttreatment patients. However, the expression of lncRNA DLEU2 expression did not alter with chemotherapy in five (13%) samples compared to matched pretreatment samples. In HER2-positive and triple-negative breast cancer subtypes, lncRNA DLEU2 expression was highly detected in the progressive, stable, and partial treatment response groups, and lowest in patients who had a complete response to the treatment (Fig. 1D). A statistically significant difference in treatment outcome was found between treatment-responsive and non-responsive patients (Fig. 1E; *P* < 0.01). Furthermore, lncRNA DLEU2 expression was significantly lower in the pathological complete response (pCR) group than in the non-pCR group (Fig. 1F; *P* < 0.01). Given the small number of patients, we used the PubMed Gene Expression Omnibus (GEO) database (GSE87455) to validate our findings for patients with breast tumors. These patients had four to six cycles of epirubicin combined with docetaxel and bevacizumab [3]. Forty-one (41/66; 62%) patients who were treated with chemotherapy had higher levels of lncRNA DLEU2 expression than the paired pre-treatment tumor samples (Fig. 1G, *P* = 0.022). Kaplan–Meier survival analysis revealed that patients with lncRNA DLEU2-high tumors had shorter disease-free survival relative to those with low-lncRNA DLEU2 (Fig. 1H; log-rank *P* = 0.013). A multivariate analysis was performed to determine the prognostic value of lncRNA DLEU2 adjusted with other clinical factors. There was a statistically significant difference in the hazard ratio (HR) between high and low lncRNA DLEU2 expression (Fig. 1I; HR 5.04, 95% Confidence interval [CI]:1.0–25.3; *P* < 0.05). These findings suggest that in BC patients, high levels of lncRNA DLEU2 expression may predict a poor therapeutic and clinical outcome.

### ROR1 and cancer stem cell (CSC) markers are highly expressed in tumors with high lncRNA DLEU2 group

**Fig. 2:**
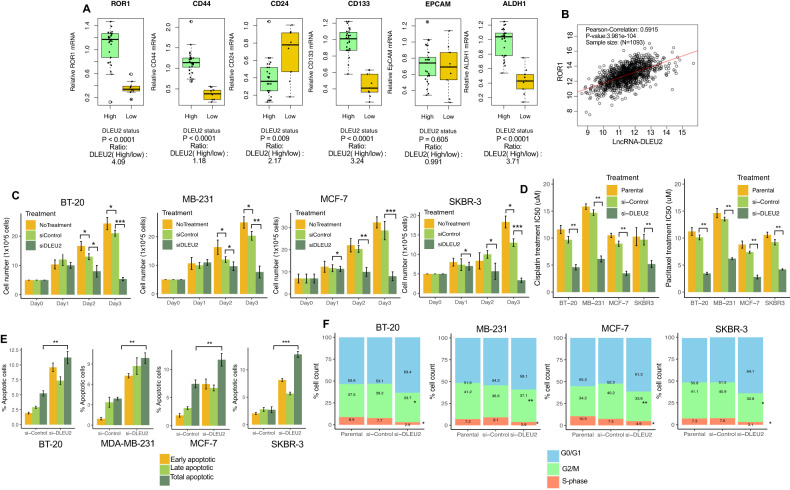
ROR1 and cancer stem cell (CSC) markers are highly expressed in the lncRNA DLEU2-high group and inhibition of lncRNA DLEU2 sensitizes BC cells to chemotherapeutic agents. **A** Comparing the expression of CSC-specific genes between lncRNA DLEU2-high and -low groups of breast cancer patients (*n* = 38). The lncRNA DLEU2-high group was defined as tumors with a lncRNA DLEU2 expression value higher than the median value for the entire samples analyzed. The remaining tumors are termed as a low-expression group. The boxes refer to the interquartile range (25–75th), and a horizontal line inside each box indicates the median. The *P* values were calculated using the Wilcoxon signed rank test. **B** Correlation between lncRNA DLEU2 and ROR1 expression in breast tumors (The GEPIA database was used to retrieve and analyze the data). **C** Cell proliferation was assessed and compared between si-lncRNA-DLEU2 and si-control in breast cancer cell lines on day 3. **D** IC50 of two chemotherapeutic agents, cisplatin and paclitaxel was analyzed and compared in parental, si-control, and si-lncRNA DLEU2-treated breast cancer cells. **E** Apoptotic assay. Analysis of cell apoptosis in breast cancer cells after treatment with si-control and si-lncRNA DLEU2. Total apoptotic cell numbers are defined as the total number of early and late apoptotic cells as retrieved from flow cytometry analysis. **F** Cell cycle analysis. Three cell-cycle phases were analyzed and compared after the treatment of cells with si-control and si-lncRNA DLEU2. *P* value was calculated for figures **B**–**E** using the Student’s t-test. *P < 0.05, **P < 0.01, ***P < 0.001.

Cancer stem cells (CSCs) play distinctive roles in chemotherapeutic resistance and have a unique ability for self-renewal features [21]. To elucidate the possible link between lncRNA DLEU2 and CSC-related genes, we first grouped all pretreated patients (n = 38) based on their expression of lncRNA DLEU2 performed using qRT-PCR analysis. Samples were grouped as lncRNA DLEU2-high if the expression of the lncRNA DLEU2 was higher than the median in all samples, on the other hand, lncRNA DLEU2-low group was segregated if the expression was lower than the median. Using these criteria, we compared the expression pattern of the stemness-associated marker ROR1 [22, 23] and several other widely recognized CSC markers between the lncRNA DLEU2-high and -low groups. A significant increase in ROR1 (*P* < 0.0001, expression ratio high/low 4.09), CD44 (*P* < 0.0001, expression ratio high/low 1.18), CD133 (*P* < 0.0001, expression ratio high/low 3.24), and ALDH1 (*P* < 0.0001, expression ratio high/low 3.71) was noted in the lncRNA DLEU2-high group compared to the lncRNA DLEU2-low expressing group (Fig. 2A). However, CD24 (*P* = 0.009, expression ratio high/low 2.17) increased in the lncRNA-DLEU2-low group (Fig. 2A). There were no statistically significant differences in EpCAM expression between the lncRNA DLEU2-high and -low groups (Fig. 2A). Our findings are supported by findings from The Cancer Genomics Atlas (TCGA) breast cancer data on BC patients and PubMed Gene Expression Omnibus (GEO) database (accession no. GSE1456) (Fig. S2A, B). Additionally, it was shown that lncRNA DLEU2 is highly positively associated with ROR1 in the LinkedOmics database (sample size: 1093; *R* = 0.59, *P* < 0.0001) (Fig. 2B). All of these findings point to an association between the expression of the lncRNA DLEU2 and ROR1 and CSCs in BC tumors and these associations possibly drive cancer cells toward chemoresistance.

### Inhibition of lncRNA DLEU2 with specific siRNA inhibits BC cell proliferation followed by enhanced chemosensitivity

To explore the mechanistic properties of lncRNA DLEU2 in BC cells, first, we evaluated how inhibiting by si-lncRNA DLEU2 altered lncRNA DLEU2 expression in BC cells (Fig. S2C). Using siRNA, we found that si-lncRNA DLEU2 significantly decreased cell proliferation as compared to si-control and parental cells in all BC cells (Fig. 2C). In si-lncRNA DLEU2 cells, cell numbers decreased from day 2 and day 3. In contrast to untreated and si-control cells, si-lncRNA DLEU2 treatment reduced cell proliferation by 45–50% in MCF-7, 35–60% in MB-231, 40–55% in BT-20, and 35–65% in SKBR3 cells in day 2 and 3 (Fig. 2C). Next, we assessed the chemosensitive properties of these cells after treating them with si-lncRNA DLEU2 and two chemotherapeutic agents. We treated BC cells with cisplatin and paclitaxel, which are commonly used chemotherapeutic agents for BC patients. In congruence with the cell growth assays, si-lncRNA DLEU2-treated cells significantly decreased their cisplatin resistance compared to si-control (Fig. 2D; Fig. S2D). Significant differences were seen between si-lncRNA DLEU2 and si-control cells when comparing the IC50 values (Fig. 2D). These findings indicate that lncRNA DLEU2 inhibition affects cell growth and enhances the chemosensitivity of BC cells.

### LncRNA DLEU2 inhibits apoptosis and alters the cell cycle in BC cells

As shown in Fig. 2C, D, si-lncRNA DLEU2-treated cells inhibited cell proliferation, raising the possibility that these inhibitions were brought about by changes to the cellular apoptosis and cell cycle alterations. To elucidate the inhibitory mechanisms, we performed an apoptosis assay on these cells, and a representative of the acquisition of cell cycle and apoptotic cell analysis is presented in Fig. S2E, F for the MB-231 cell line. We found that si-lncRNA DLEU2 significantly induced apoptotic cell numbers when compared to si-control treated cells (Fig. 2E). Additionally, flow cytometry analysis of lncRNA DLEU2 depleted cells detected a modest increase in the number of the G0-G1 population and decreased the number of S-phase cells (Fig. 2F). These results imply that the abatement of cell growth, viability, and chemosensitivity is primarily due to cell death and inhibition of cell cycle progression.

### LncRNA DLEU2 expression is correlated with the expression of EMT-related genes

**Fig. 3:**
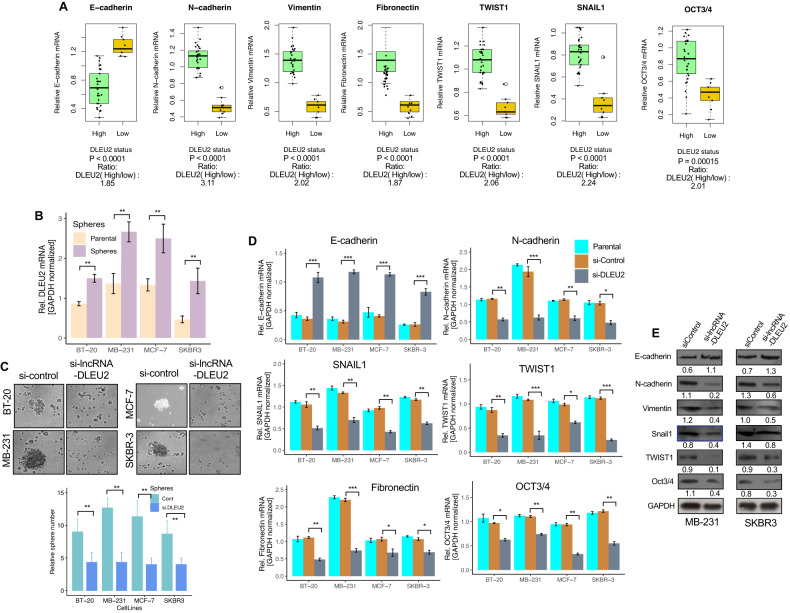
LncRNA DLEU2 expression is correlated with EMT-related gene expression. **A** Analysis, and comparison of the expression of EMT-related genes between lncRNA DLEU2-high and-low groups (*n* = 38). The lncRNA DLEU2 -high group was defined as tumors with a lncRNA DLEU2 expression value higher than the median value for the entire samples analyzed. The remaining tumors are termed, low-expression groups. The boxes refer to the interquartile range (25–75th), and a horizontal line inside each box indicates the median. The *P* values were calculated using the Wilcoxon signed rank test. **B** LncRNA DLEU2 expression level was compared between parental and sphere cells using qRT-PCR. Student *t-test* was used for calculating the *P* value **P < 0.01. **C** Morphology, and quantification of spheres cultured under the treatment of si-control and si-lncRNA DLEU2. The bar graph (right panel) shows the quantification of sphere growth. *P* value was calculated using the Student *t*-test. **P < 0.01. **D** The expression of EMT-specific genes in parental, si-control, and si-lncRNA DLEU2-treated breast cancer cells measured by qRT-PCR. The difference between si-control and si-lncRNA DLEU2 was analyzed. Student *t-test* was used for calculating the *P* value. *P < 0.05, **P < 0.01, ***P < 0.001. **E** Western blot analysis of EMT-related genes in si-control, si-lncRNA DLEU2 MB-231, and SKBR3 cells.

Cancer cells have been shown to acquire stemness features in response to chemotherapy [24], and targeting CSC pathways that induce epithelial-to-mesenchymal transition (EMT) may be more effective than those strategies that only target CSCs. To examine the relevance and alternative mechanisms of lncRNA DLEU2 and BC cell properties, first, we elucidated the relationship between lncRNA DLEU2 and EMT-related gene expression. We compared the expression of genes associated with EMT in the lncRNA DLEU2-high and -low groups. To this end, we used the PubMed GEO database accession no. GSE1456 on BC patients. Our analysis of the GSE1456 data revealed that the expression of EMT-related genes differed significantly between high and low levels of the lncRNA-DLEU2 (Fig. S3A). The TCGA breast dataset shows comparable expression differences between high and low levels of the incRNA DLEU2 (Fig. S3B). We then evaluated the expression of EMT-related genes in the lncRNA DLEU2-high and -low groups to verify the results of the public dataset. We found that the majority of the lncRNA DLEU2-high cell populations co-express genes associated with EMT. Compared to the high lncRNA DLEU2 group, the lncRNA DLEU2-low group showed a considerably higher expression of epithelial marker E-cadherin (Fig. 3A, *P* < 0.0001, expression ratio high/low 1.85). The expression of the mesenchymal markers, i.e. N-cadherin (*P* < 0.0001), vimentin (*P* < 0.0001), and fibronectin (*P* < 0.0001), on the other hand, were all significantly increased in the lncRNA DLEU2-high group compared to the -low group (Fig. 3A). Furthermore, the expression of mesenchymal markers N-cadherin was 3.11 times higher in the lncRNA DLEU2-high group than in the low group. Similarly, vimentin (expression ratio: 2.02), and fibronectin (expression ratio:1.87) were both two times higher than the low group. TWIST1 (expression ratio high/low 2.06), and SNAIL1 (expression ratio high/low 2.24) expression were also significantly increased in the lncRNA DLEU2-high group. Likewise, stemness marker OCT3/4 expression was significantly higher in the lncRNA DLEU2-high group compared to the low group (Fig. 3A; *P* < 0.001, expression ratio high/low 2.01). These results support the view that the lncRNA DLEU2 is associated with EMT in BC and drives cells toward metastatic and invasive characteristics and chemotherapeutic resistance.

To confirm the patient’s dataset in vitro, we used four BC cell lines for sphere formation assays and generated spheres. The sphere assay potentially induces the enrichment of CSCs and EMT-related gene expression [25]. In all cell lines, lncRNA DLEU2 mRNA expression was significantly increased in spheres, at least 1.5–2.5-fold higher compared to parental cells (Fig. 3B). Furthermore, silencing lncRNA DLEU2 significantly reduced the sphere formation efficiency (Fig. 3C), suggesting that lncRNA DLEU2 expression may have the ability to increase the stemness ability by inducing CSCs and EMT-related genes in BC. To confirm this, we evaluated whether silencing of lncRNA DLEU2 might have any effects on EMT-related gene expression. Compared to si-control cells, si-lncRNA DLEU2 cells had increased E-cadherin mRNA expression in MCF-7 cells. On the other hand, silencing of lncRNA DLEU2 also significantly decreased N-cadherin and fibronectin expression in all cell lines, with the strongest reduction seen in MCF-7 and SKBR3 cells (Fig. 3D). In addition, TWIST1, SNAIL1, and OCT3/4 also decreased in si-lncRNA DLEU2 cells (Fig. 3D). We have investigated whether lncRNA DLEU2 knockdown inhibits EMT and stem cell markers at the protein level. At the protein level as assessed by western blot, elevated E-cadherin expression was found in MB-231 and SKBR3 cells treated with si-lncRNA DLEU2 (Fig. 3E). In addition, N-cadherin, TWIST1, and Oct3/4 expression decreased in both cell lines treated with si-lncRNA DLEU2 (Fig. 3E). These results suggest that lncRNA DLEU2 regulates EMT and stemness in BC.

### LncRNA DLEU2 specifically promotes ROR1 expression in chemoresistant BC cells

**Fig. 4:**
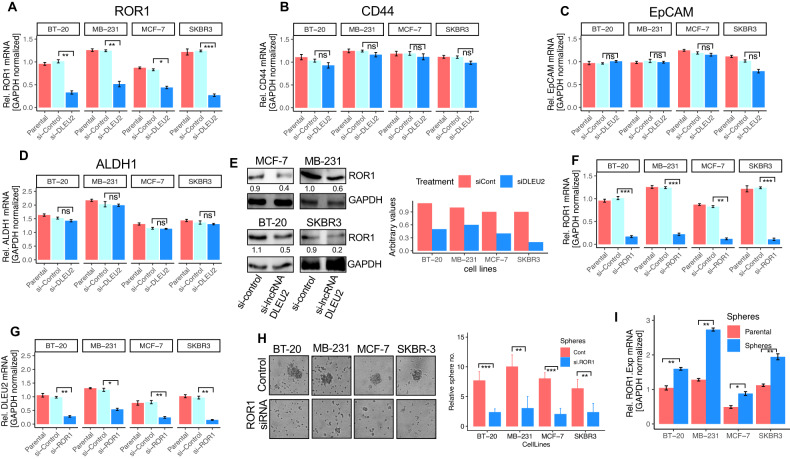
ROR1 expression in si-lncRNA DLEU2-treated cells and lncRNA DLEU2 expression in si-ROR1 treated cells. **A** Analysis of ROR1 expression in parental, si-control, and si-lncRNA DLEU2-treated cells. mRNA expression was analyzed by qRT-PCR. The differences in expression were compared between si-control and si-lncRNA DLEU2-treated cells. Student *t-test* was used for calculating the *P* value. *P < 0.05, **P < 0.01, ***P < 0.001. Cancer stem cell-related genes expression **B** CD44, **C** EpCAM, and **D** ALDH1 in parental, si-control, and si-lncRNA DLEU2 cells. The expression of each marker was analyzed by qRT-PCR. The differences in expression were analyzed between si-control and si-lncRNA DLEU2-treated cells. Student *t-*test was used for calculating the *P* value. *ns*-not significant. **E** Western blots of ROR1 protein expression in si-lncRNA DLEU2 and si-control treated breast cancer cells with the quantification for each cell line. **F** ROR1 expression in parental, si-control, and si-ROR1 cells. mRNA expression was analyzed by qRT-PCR. The differences in expression were compared between si-control and si-ROR1 treated cells. *P* values were calculated using the Student *t-*tes*t*. **P < 0.01, ***P < 0.001. **G** LncRNA DLEU2 expression in parental, si-control, and si-ROR1 cells. qRT-PCR was used to analyze the mRNA expression. The differences are compared between si-control and si-ROR1 treated cells. Student *t-test* was used for calculating the *P* value. *P < 0.05, **P < 0.01. **H** Morphology of spheres cultured under the treatment of si-control and si-ROR1. The bar graph (right panel) shows the relative number of spheres generated for each cell line in control and si-ROR1 treated groups. *P* values were calculated using the Student t-test. **P < 0.01, ***P < 0.001. **I** The ROR1 mRNA expression was analyzed between parental and sphere cells using qRT-PCR. Student *t-*test was used for calculating the *P* value. *P < 0.05, **P < 0.01.

Our patient’s data as well as GEO data analysis showed that lncRNA DLEU2-high cells exhibited higher ROR1 expression along with several CSC markers (Fig. 2A; Fig. S2A). Previously, it has been shown that BC cells with high expression of ROR1 demonstrate poorly differentiated characteristics and are associated with EMT and stemness [14, 22]. As a result, we aim to investigate whether ROR1 was specifically targeted by lncRNA DLEU2. Thus, the mRNA expression of CSC markers including ROR1 was compared between si-control and si-lncRNA DLEU2 in BC cell lines. Figure 4A indicates that there are significant differences in the ROR1 mRNA expression between si-control and si-lncRNA DLEU2 cells. Except for ROR1, however, no significant differences in mRNA expression of CSC markers CD44, EpCAM, or ALDH1 were seen between si-control and si-lncRNA DLEU2 (Fig. 4A–D). By contrast, in SKBR3 cells, the expression of CSC-related genes differed significantly between si-control and si-lncRNA DLEU2-treated cells (Fig. 4A, *P* < 0.001). In all of the cell lines evaluated, treatment with si-lncRNA DLEU2 had no visible effect on the expression of CD44, EpCAM, or ALDH (Fig. 4B, C). Further to that, si-lncRNA DLEU2 inhibition decreased the expression of the ROR1 protein in all cell lines (Fig. 4E). As a result, we speculate that ROR1 expression is exclusively linked with the lncRNA DLEU2 expression.

### ROR1 inhibition decreases lncRNA DLEU2 expression and sphere formation ability in BC cells

Next, ROR1 was silenced in BC cells to identify if it had any impact on the expression of the lncRNA DLEU2. Silencing ROR1 significantly decreased its expression concomitant with a decrease in lncRNA DLEU2 mRNA expression in all cell lines (Fig. 4F, G; Fig. S4). These results indicate that ROR1 also reciprocally regulates lncRNA DLEU2 expression in BC cells. Furthermore, we used the sphere formation assay and generated spheres to investigate whether ROR1 inhibition attenuates the self-renewal capacity of BC cells. Inhibition of ROR1 also inhibited the sphere formation efficiency (Fig. 4H). Additionally, ROR1 mRNA expression was at least 1.5–2.5-fold higher in the spheres in all cell lines to parental cells (Fig. 4I), suggesting that lncRNA-DLEU2-ROR1 expression may have the ability to increase the sphere formation ability by inducing CSC-related genes in BC. This raises the possibility that ROR1 and lncRNA DLEU2 cooperatively act in therapeutic resistance by inducing EMT and CSCs.

### ROR1 expression is associated with EMT-related gene regulation in BC cells

**Fig. 5:**
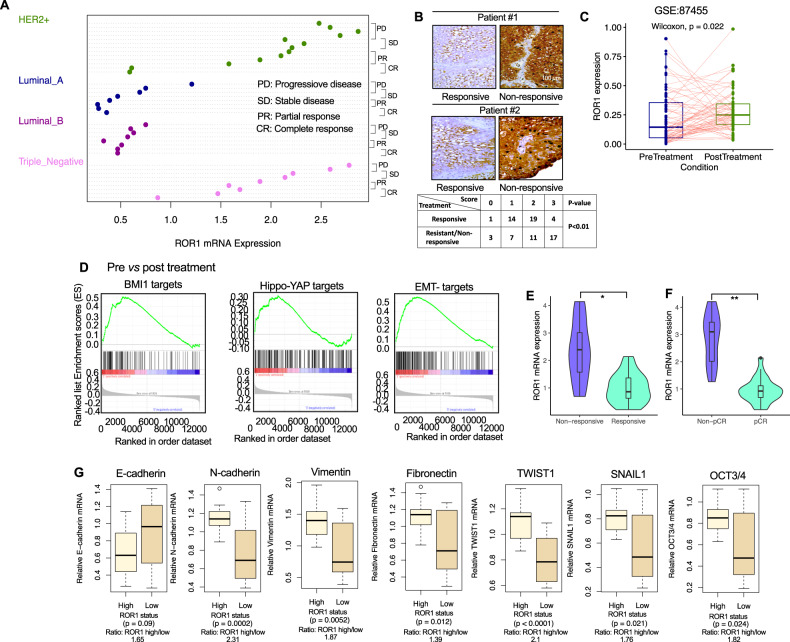
ROR1 inhibition decreases lncRNA DLEU2 expression and sphere formation ability in breast cancer cells. **A** The ROR1 mRNA expression in different breast cancer molecular subtypes and response to chemotherapy. Complete response (CR), partial response (PR), stable disease (SD), and progressive disease (PD) in breast cancer patients (*n* = 38). Patients were treated with neoadjuvant chemotherapy as described in the materials and methods section. **B** Immunohistochemical staining of ROR1 in breast tissues received from breast cancer patients (*n* = 38) in treatment-responsive and resistant patients. These patients were treated with docetaxel/epirubicin with/without cyclophosphamide. Scale bar 100 μm. The table below shows the elevated expression of ROR1 of the breast cancer clinical specimens obtained from patients after chemotherapy. Fisher’s exact test was used to test the significance. **C** ROR1 expression in pre- and post-treatment breast cancer patients treated with four cycles of epirubicin combined with chemotherapy (Wilcoxon test, *P* = 0.022). GSE84755, Kimbung et al. [3]. **D** Enrichment plots of genes associated with the activation of BMI1, Hippo-YAP, and EMT as assessed by RNA-sequencing (GSE84755; Kimbung et al. [3]) on pre-and post-treatment. **E** Violin plot shows the relative ROR1 expression levels in chemotherapy-responsive and resistant breast cancer patients (*P < 0.05). **F** Violin plot shows the expression of ROR1 in breast cancer patients with pCR (pathological complete response) compared with those of non-pCR patient groups (**P < 0.01). **G** Comparison of epithelial-to-mesenchymal (EMT) and stemness-related gene expression between ROR-high and -low groups (n = 38). ROR1 -high group was defined as tumors with a ROR1 expression value higher than the median for all samples. The boxes represent the interquartile range (25th to 75th), and the horizontal lines inside the boxes indicate the median. Whiskers indicate the minimum and maximum values. *P* values were calculated using the Student’s *t-test*.

We have shown that the lncRNA DLEU2-high cell population had higher ROR1 mRNA (Fig. 2A, Fig. S2A) and that silencing lncRNA DLEU2 attenuated ROR1 mRNA (Fig. 4A), indicating a potential crosstalk between the two genes. We thus posited that ROR1, like lncRNA DLEU2, is intricately linked to the EMT mechanism. To determine the expression of ROR1 mRNA in different molecular subtypes and the effectiveness of therapy, we obtained biopsy tissues from BC patients (*n* = 38) from those described in Fig. 1 (Supplementary Table S2). Surprisingly, the ROR1 mRNA was highly expressed in HER2+ and triple-negative breast cancer (TNBC) tumors primarily in those patients who had a progressive, stable, or partial response to treatment (Fig. 5A). Furthermore, ROR1 protein was also detected on either ER+ (estrogen receptor-positive) or ER- (ER-negative) BC tissues. In matched BC tissues, we found that 21 (55%) of 38 matched tumors had higher ROR1 expression (Fig. 5B; Supplementary Table S2). Thirteen (34% of 38) BC tumors showed no changes in ROR1 staining intensity after treatment. Compared to matching pretreatment tissue, the expression of ROR1 was reduced in four (11%) after treatment (Supplementary Table S2). To validate our results, we analyzed the GEO database (accession no. GSE87455) as shown in Fig. 1C and F. Forty-four (44/66; 67%) tumors exhibited higher expression of ROR1 than the paired pre-treatment tumor samples (Fig. 5C). Furthermore, gene set enrichment analysis revealed that compared to paired pre-treatment tumors, post-treatment BC patients’ tumors exhibited increased expression of genes linked to the Hippo-YAP/TAZ, BMI1 and EMT (Fig. 5D).

Next, we assessed the ROR1 mRNA level in patients who responded to treatment and cases who did not, as well as in cases with non-pCR and pCR. Patients who responded to therapy and those who did not showed statistically significantly different levels of ROR1 expression (Fig. 5E, *P* < 0.05). Moreover, pCR patients had significantly lower than that in the non-pCR group (Fig. 5F, *P* < 0.002). These findings imply that ROR1 expression promotes treatment resistance and therefore plays a role in disease recurrence. These findings further raise the possibility of close interaction between the ROR1 and EMT-related genes. As a result, we assessed and compared the EMT-related markers between groups that had high ROR1 and those that had low ROR1 in all pretreatment patients (n = 38). N-cadherin (*P* = 0.0002), fibronectin (*P* = 0.012), and vimentin (*P* = 0.0052) all had significantly higher levels in the ROR1-high group compared to the ROR1-low group, despite the fact that E-cadherin (*P* = 0.09) expression was, as expected considerably higher in the ROR-low group (Fig. 5G). Additionally, the ROR1-high group had substantially higher levels of TWIST1 (*P* < 0.0001), SNAIL1 (*P* = 0.024), and OCT3/4 (*P* = 0.021) (Fig. 5G). We further assessed and confirmed our results from a PubMed Gene Expression Omnibus (GEO) database (GEO accession no. GSE1456). Figure S5A shows the expression of all EMT-related genes and OCT3/4 with their expression ratios. As noted, a significant decrease in E-cadherin was found in the ROR1-high group compared with the ROR1-low group (*P* = 0.048; ROR1-High/low ratio1.71). All EMT-related genes, such as N-cadherin (*P* < 0.0001, expression ratio 1.29), vimentin (*P* < 0.0001, expression ratio 2.29), TWIST1 (*P* < 0.001; expression ratio 1.44) were upregulated in the ROR1-high group (Fig. S5A). However, there were no significant changes in the expression of fibronectin between the ROR1-high and -low groups (*P* = 0.221; expression ratio 1.05). In addition, the stemness marker OCT3/4 was significantly enhanced in the ROR1-high group compared with the low group (*P* < 0.001; expression ratio 4.43). Furthermore, TCGA breast cancer data show similar expression of EMT markers between ROR1-high and -low groups (Fig. S5B).

**Fig. 6:**
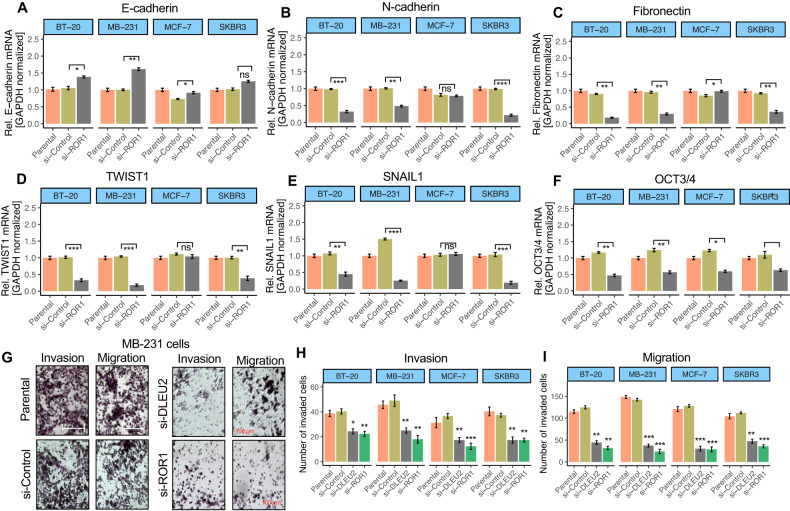
The ROR1 expression correlates with the expression of EMT-related genes in breast cancer cells. **A**–**F** Analysis of the expression of EMT and stemness-related genes between parental, si-control, and si-ROR1 cells. The expression difference between si-control and si-ROR1 are compared for statistical significance. *P* values were determined using Student’s *t*-test. *P < 0.05, **P < 0.01, ***P < 0.001. **G** Representative images of cell invasion and migration of MB-231 cells in si-control and si-ROR1 treated cells. Scale bar 100 μm. Invasion (**H**), and migration (**I**) assay in parental, si-control, si-ROR1, and si-lncRNA DLEU2 cells. The invasion and migration index was calculated by dividing the total number of parental cells that invaded or migrated through the chambers. The differences between si-control and si-ROR1 or si-lncRNA DLEU2 were compared for statistical significance. The student’s *t*-test was used for significance analysis. *P < 0.05, **P < 0.01, ***P < 0.001.

Next, the relationship between ROR1 and genes associated with EMT was investigated in vitro using BC cell lines. Figure 6A shows that E-cadherin was significantly increased in si-ROR1 treated BT-20, MB-231, and MCF-7 cells, while it was unchanged in SKBR3 cells. On the other hand, N-cadherin, fibronectin (FN1), TWIST1, SNAIL1, and Oct3/4 dramatically downregulated in si-ROR1 cells compared to si-control treated cells (Fig. 6B–F). To assess the phenotypic changes of these cells, we performed invasion and migration assays. si-ROR1 and si-lncRNA DLEU2 treatment resulted in inconsiderably fewer migrated and invaded cells (Fig. 6G–I), indicating that the lncRNA DLEU2/ROR1 pathway plays a pivotal role in the induction of EMT and invasive behaviors in BC.

### ROR1 expression is associated with cancer stem cell-related gene expression in BC cells

**Fig. 7:**
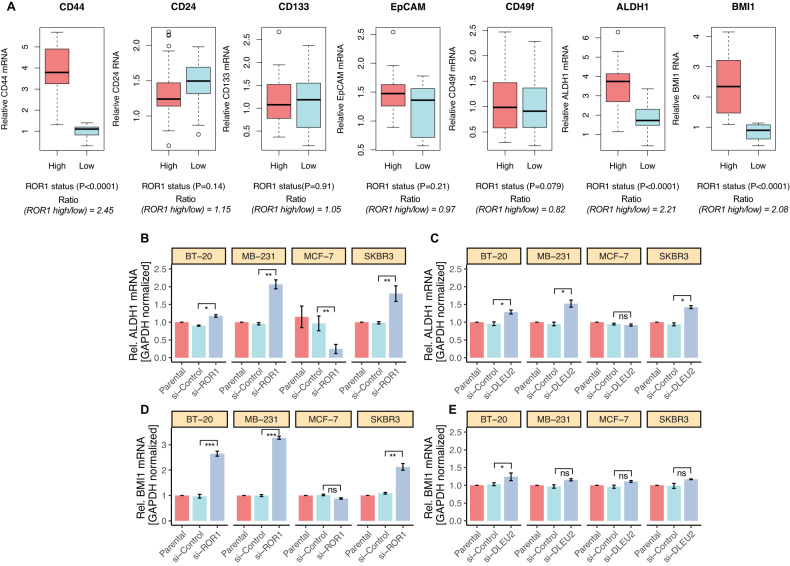
The ROR1 expression correlated with the expression of cancer stem cell-related genes in breast cancer cells. **A** Comparison of breast cancer stem cell-related genes between ROR1-high and -low groups (*n* = 38). The boxes represent the interquartile range (25th to 75th), and the horizontal lines inside the boxplot indicate the median. Whiskers on top and bottom indicate the maximum and minimum values of the data. The ROR1-high group was defined as tumors with an ROR1 expression value higher than the median for all samples. The ratios were calculated by dividing the mRNA expression value of the high group by the expression value of the low group. *P* values were calculated using the Wilcoxon signed rank test. ALDH1 expression in **B** si-ROR1, and **C** si-lncRNA DLEU2 cells. The expression differences between si-control and si-ROR1 and si-lncRNA DLEU2 were compared. *P* value was calculated using Student’s *t-*test. The BMI1 mRNA level in parental, si-control, and si-ROR1 (**D**) and si-lncRNA DLEU2 (**E**) The expression difference between si-control, si-ROR1, and si-lncRNA DLEU2 were compared. *P* value was calculated using Student’s *t-*test.

It has been reported that ROR1 signaling may regulate the maintenance, self-renewal, and drug resistance in breast and other cancers [13, 22]. Moreover, in BC, CD44, ALDH1, and BMI1 are defined as CSC markers [26]. In light of the potential interaction between ROR1 and CSC, we evaluated and compared the expression of several well-known and well-defined CSC markers between ROR1-high and -low groups in all pretreatment patients (*n* = 38) BC tissues. We have found a significant difference in the expression of CD44, ALDH1, and BMI1 between ROR1-high and -low groups (Fig. 7A). For instance, the expression of CD44 increased significantly in the ROR1-high group but not in the ROR1-low group (Fig. 7A; *P* < 0.0001; expression ratio 2.45). In contrast, CD24 expression was higher in ROR1-low groups compared to ROR1-high groups, however, there was no statistically significant difference (Fig. 7A; *P* = 0.14, expression ratio 1.15). Similarly, there was no discernible difference between the ROR1-high and -low groups in the expression of CD133, EpCAM, and CD49f (Fig. 7A). On the other hand, the expression of ALDH1 (*P* < 0.0001, expression ratio 2.21) and BMI1 (*P* < 0.0001; expression ratio 2.08) was significantly higher in the ROR1-high group compared to the -low group (Fig. 7A). These findings suggest that the CSC pathway was selectively elevated in the ROR1-high group versus the -low group, raising the possibility that ROR1 expression is associated with CSC pathway activation, which in turn drives BC cells to treatment resistance.

To validate our results, we used the GEO database on breast tumors with accession number GSE1456. Using this dataset, we ascertained the expression of CSC-related genes between ROR1-high and -low groups. The ratio between the ROR1-high and -low groups was 1.68, and the expression of CD44 was significantly higher in the ROR1-high group compared to the low group (*P* < 0.001) (Fig. S6A). EpCAM, a CSC marker, expressed only in the ROR1-low group (P < 0.0001) with an expression ratio of 1.31 (Fig. S6A). There were no discernible differences in the expression of the three prominent breast cancer CSC markers, CD24 (*P* = 0.066; expression ratio: 0.973), CD133 (*P* = 0.670; expression ratio:1.02), and CD49f (*P* = 0.431; expression ratio: 0.907) between the ROR1-high and -low groups (Fig. S6A). It is interesting to note that the ALDH1 (*P* < 0.0001; expression ratio 2.28) and BMI1 (*P* < 0.001; expression ratio:1.38) expression levels were significantly higher in the ROR1-high group compared to the -low group (Fig. S6A). These findings further raise the possibility that ROR1 expression drives BC cells towards stemness and may potentially play a role in therapeutic resistance. Additionally, the TCGA breast cancer data validate the above results (Fig. S6B).

According to the result presented in Fig. S6A, it indicates that ALDH1 and BMI1 expression in the ROR1-high group was 3.8- and 3.3-fold higher than in the low group. Additionally, we illustrate the association between lncRNA DLEU2 expression and CSC markers expression (Fig. 2A). As such, we reasoned that there could be a positive association between the ROR1/lncRNA DLEU2 pathway activation and ALDH1 and BMI1 expression. We employed four BC cell lines to elucidate the association between ROR1 and lncRNA DLEU2 as well as the possible interaction between ALDH1 and BMI1. After treating the cells with si-ROR1 and si-lncRNA DLEU2 the expression of ALDH1 and BMI1 was determined using qRT-PCR. ALDH1 expression was significantly increased in BT-20, MB-231, and SKBR3 cells but not in MCF-7 cells (Fig. 7B, C). On the other hand, treatment of cells with si-lncRNA DLEU2 produced comparable effects as seen in Fig. 7B, suggesting that ALDH1 regulates ROR1/lncRNA DLEU2 pathway activity in BC cells. The relationship between ROR1/lncRNA DLEU2 and BMI1 was next determined in vitro, and the BMI1 mRNA level was assessed by qRT-PCR after cells were treated with si-ROR1 and si-lncRNA DLEU2. Although si-ROR1 increased BMI1 expression significantly in BT-20, MB-231, and SKBR3 cells, it did not increase BMI1 expression in MCF-7 cells (Fig. 7D, E). However, si-lncRNA DLEU2 treatment had no effect on BMI1 expression except for BT-20 cells. These results suggest that BMI1 potentially regulates the ROR1 pathway in BC.

### TGF-β induces ROR1-dependent BMI1 activation

**Fig. 8:**
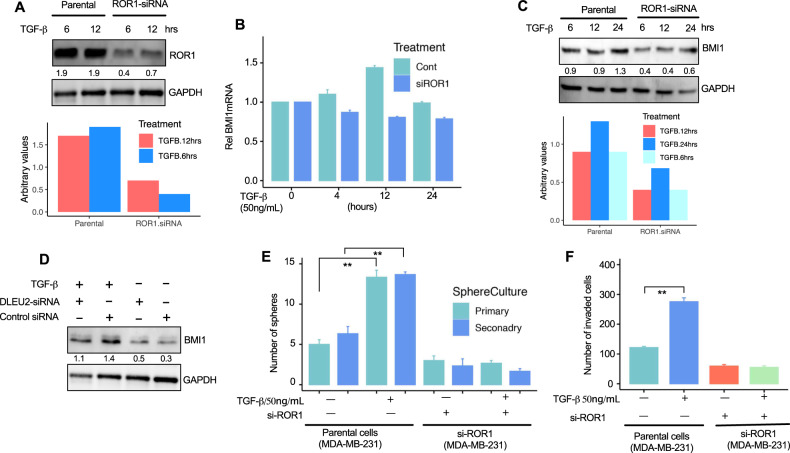
TGF-β induces ROR1-dependent BMI1 activation. **A** Immunoblot analysis of ROR1 protein using lysates from parental and si-ROR1 treated MB-231 cells that were stimulated with TGF-β for the indicated time with the quantification results below. **B** BMI1 mRNA levels in MB-231 cells treated with TGF-β at 50 ng/mL and si-control and si-ROR1 at an indicated time interval were examined by quantitative PCR (qPCR). The figure shows the mean expression of BMI1 compared to time “0”. Experiments were performed in triplicate unless indicated otherwise and normalized with GAPDH. The error bars indicate the ±SEM. **C** Immunoblot analyses of BMI1 protein using lysates from parental and si-ROR1 treated MB-231 cells that were stimulated with TGF-β for the indicated times and quantification results below. **D** Immunoblot analyses of BMI1 protein using lysates from parental and si-control and si-lncRNA DLEU2-treated MB-231 cells that were stimulated with TGF-β. **E** The bar graph indicates the average number of spheroids formed by parental or si-ROR1 cells treated with or without TGF-β in triplicate ±SEM. *P* values were calculated using a two-tailed paired Student’s *t*-test. **P < 0.01) **F** The bar graph indicates the average number of invaded cells from parental or si-ROR1 cells treated with or without TGF-beta in triplicate ±SEM. *P* values were calculated using a two-tailed paired Student’s *t-*tes*t*. **P < 0.01).

Finally, we tested whether exogenous TGF-β might activate BMI1 in BC cells in this unique signaling context, finding that ROR1 silencing significantly impaired the CSCs and EMT activity in BC cells (Figs. 4 and 6). To get more insight into this, we knocked down ROR1 *via* siRNA in MB-231 cells after cells were treated with TGF-β (Fig. 8A). Treatment with exogenous TGF-β for 6, 12, and 24 h gradually enhanced the expression of BMI1 mRNA and protein in parental MB-231 cells but not in the si-ROR1 treated cells (Fig. 8B, C), suggesting that TGF-β is capable of inducing BMI1 and subsequently increasing the CSCs and stemness activity. We further examine whether TGF-β could have any influence on BMI1 under the treatment of si-lncRNA DLEU2. Treatment of parental MB-231 with si-lncRNA DLEU2 impaired the strength of TGF-β to enhance BMI1 protein expression (Fig. 8D). To characterize the functional consequences of si-ROR1 treated cells, we performed in vitro spheroids and invasion assays. Cells treated with si-ROR1 formed fewer spheroids than control cells (Fig. 8E). Furthermore, treatment with exogenous TGF-β enhanced the invasion capacity of cells in MB-231 control cells but not in si-ROR1 knockdown cells (Fig. 8F).

## Discussion

Because of large-scale OMICS research, our understanding of the roles of lncRNAs in regulating EMT and CSCs in breast carcinogenesis is rapidly expanding. CSCs are responsible for tumor metastasis and recurrence due to their self-renewal characteristics and ability to initiate tumorigenesis [27]. Many studies have reported the close associations between lncRNAs and BC progression and metastasis [28, 29]. Recent studies have reported that lncRNAs play a pivotal role in cell fate determination, reprogramming, and deciding which proteins, genes, and chromosomes are activated or reactivated [30, 31]. In the present study, the expression of lncRNA DLEU2 and its correlation with ROR1 was analyzed using BC patient specimens and cell lines (which includes MCF-7, MB-231, BT-20, and HER2+ cells) and various bioinformatic tools, retrieved from ONCOMINE, TCGA, GEPIA, DAVID, LinkedOmics CCLE, and PubMed GEO databases. Our findings show a remarkable association between lncRNA DLEU2 and ROR1 expression, which together play a role in EMT and therapeutic resistance by inducing CSCs in BC patients undergoing chemotherapy.

LncRNA DLEU2 has been identified as a tumor suppressor RNA in several cancers, implying that it could be used as a molecular biomarker for diagnosis and treatment [8]. Furthermore, abnormal expression of lncRNA DLEU2 plays a crucial role in tumor progression and metastasis in pancreatic, lung, and hematopoietic malignancies [9–11], suggesting that lncRNA DLEU2 is involved in diverse cancers with similar functions. Additionally, lncRNA DLEU2 is also known to promote the occurrence of laryngeal, lung, and hepatic cancer [32, 33]. However, in BC, the precise role and function of lncRNA DLEU2 is unknown. This is the first study to use bioinformatics analyses and public databases such as ONCOMINE, GEPIA, and GEO datasets and in vitro assays to link the lncRNA DLEU2 to EMT and CSC phenotypes in BC. Our results showed that lncRNA DLEU2 expression was higher in tumor tissues compared to disease-free breast samples. In addition, the CCLE and EBL-EBI databases showed similar expression patterns in BC cell lines. Furthermore, a high level of lncRNA DLEU2 was associated with a shorter PFS, suggesting that lncRNA DLEU2 could serve as a prognostic biomarker for BC. Moreover, CSCs and EMT-related genes are the most significantly correlated with lncRNA DLEU2 expression. All CSC- and EMT-related genes are 1.5-to-2.4-fold higher in lncRNA DLEU2 -high cells than in the lncRNA DLEU2-low expressed BC cells.

To establish our hypothesis, we analyzed putative mechanisms and the lncRNA DLEU2 functions in BC cells to support our findings, and we showed that lncRNA DLEU2 affected BC cell proliferation and resistance to the chemotherapeutic drug cisplatin in vitro. In line with this, we further found that the expression of lncRNA DLEU2 was highly upregulated in therapy-resistant BC patients’ tumor tissues and associated with poor DFS. In laryngeal squamous cell carcinoma, lncRNA DLEU2 affects cell proliferation and cell cycle [32]. In our analysis, we found that lncRNA DLEU2 may promote cell growth and regulate the cell cycle. Since inhibiting lncRNA DLEU2 reverses these effects, lncRNA-DLEU2 likely inhibits cell growth by regulating the cell cycle. In a recent study, Dong et al. [34] studied the contribution of lncRNA DLEU2 in the promotion of EMT in endometrial cancer. Furthermore, EMT promotes cancer migration, invasion, and metastasis and is associated with a poor prognosis. To combat cancer progression, one of the most effective strategies may be to target EMT. Consistent with these observations, we found that lncRNA DLEU2 expression was strongly correlated to the expression of EMT genes in BC specimens, which was further supported by publicly available datasets. Regardless of the BC subtypes, functionally, the suppression of lncRNA DLEU2 exhibited substantial alterations in the expression of EMT-related genes in BC cells.

Tumor-initiating cells, or CSC, are cancer cells that have the potential to repopulate the self-renewal and differentiation abilities, facilitating the primary tumors to metastasize distant organ sites [24]. On the other hand, EMT is closely related to CSC in that cells undergoing EMT can acquire stem-like characteristics, establishing an intriguing conjunction between EMT and cancer stem cells [35]. Given this tight interaction between EMT and CSCs, we have compared CSC markers between lncRNA DLEU2-high and -low groups. There were significant expression differences in CD44, CD24, CD133, ALDH1, and ROR1 between the lncRNA DLEU2-high and -low groups but not in EpCAM expression. These findings suggest that lncRNA DLEU2 regulates EMT and CSC functions in BC.

Cellular differentiation, growth, and metastasis are all significantly influenced by receptor tyrosine kinases (RTKs) [36, 37]. Many functional findings show that ROR1 is linked to non-canonical WNT-signaling to promote cancer cell survival, growth, and invasion, and ROR1 exerts cellular signaling in both kinase-dependent and independent mechanisms [38]. Given that ROR1 signaling actively contributes to the maintenance, self-renewal, and chemoresistance of cancer cells, this raises the possibility that there is a link between ROR1 and the lncRNA DLEU2 pathway and that this pathway regulates CSC behavior. However, no studies have shown an association between ROR1 and lncRNA DLEU2. Our findings lend credence to this new observation by demonstrating that lncRNA DLEU2 knockdown attenuated ROR1 expression in BC cells, restraining BC cells’ ability to proliferate, invade, migrate, and sensitize to chemotherapeutic agents.

Several studies have reported that higher expression of ROR1 in BC cells is associated with relatively rapid disease relapse and short survival [14, 22, 39]. Silencing ROR1 represses the expression of EMT-related genes and impairs cancer cells’ migration and invasion as well as their metastatic abilities, suggesting the stemness-inducing role of ROR1 in BC cells [14]. Interestingly, EMT-related genes are exclusively expressed in the ROR1-high group compared to the -low ROR1 group, implying that ROR1 regulates metastasis and therapeutic resistance in BC. To combat the EMT and CSC-related metastasis and therapeutic resistance, the anti-ROR1 mAb cirmtuzumab may be very effective in reversing the cancer stemness and treatment resistance [13]. Cirmtuzumab has currently completed a phase I clinical trial in patients with relapsed or refractory CLL, in which treatment with cirmtuzimab inhibited leukemia-cell activation of Rho-GPTPase and ROR1 signaling [23].

Overexpression of ROR1 in BC is also associated with increased levels of ALDH1 and BMI1. The TGF-β/ROR1 pathway may likely induce ALDH1 and BMI1 expression through its ability to interact with lncRNA DLEU2. Although the precise mechanisms and interactions involving lncRNA DLEU2 and ROR1 are unknown, the resulting increase in BMI1 and ALDH1 may partially explain the increased ability of BC cells with the high level of lncRNA DLEU2 and ROR1 to facilitate tumor growth, distant metastasis, or resist the chemotherapeutic treatment relative to BC cells lacking lncRNA DLEU2 and ROR1.

LncRNAs have gained phenomenal interest due to their distinct and unique roles in BC progression. While certain lncRNAs may regulate physiological functions and play a role in the development of tumors, drug resistance, and metastasis, other lncRNAs may inhibit these processes [29]. LncRNA DLEU2 and ROR1 are selectively overexpressed in the BC cells, particularly in tumors that are resistant to treatment. Both ROR1 and lncRNA DLEU2 cooperatively regulate EMT and CSC activity in BC and are inversely correlated with patient outcomes. To demonstrate how inhibition of lncRNA DLEU2 and ROR1 decreases BC cell migration, invasion, and spheroids generation while enhancing the chemosensitivity, we assessed and utilized the BC cell lines as our model. Our findings demonstrate that inhibition of the lncRNA DLEU2/ROR1 pathway may complement the antitumor or anti-metastatic activity by eliminating drug-resistant CSCs or inhibiting tumor cells from acquiring CSC-like characteristics. This strategy may serve as a guide as we develop novel therapeutic strategies for BC patients who are challenging to treat. Notably, trends in LncRNA DLEU2 and ROR1, as well as EMT and CSC markers, were detected between our patients’ samples and samples evaluated from three different publicly accessible datasets (TCGA, GEPIA, and GEO). This shows that the LncRNA DLEU2/ROR1 pathway might be a potential target for increasing chemotherapeutic sensitivity.

## Conclusions

In summary, our results revealed the differential expression of the lncRNA DLEU2 and ROR1 in BC samples. We also show the unique role of the lncRNA DLEU2/ROR1 pathway in promoting the EMT and cancer stemness behavior via a mechanism closely engaging with TGF-β signaling, which drives EMT and CSC phenotypes *via* ALDH1 and BMI1 activation. LncRNA DLEU2 is also involved in cancer cell proliferation, chemoresistance, apoptosis inhibition, and cell cycle regulation. Thus, this study advances our understanding of the underlying molecular process responsible for EMT and cancer stemness, while also providing a novel target for BC therapy, improved chemotherapy response, and increased survival of BC patients. However, further molecular and cellular analysis is yet to be performed to identify the underlying mechanisms of lncRNA DLEU2/ROR1 mediated BC progression and metastasis, which may lead to novel therapeutic strategies and predictive and prognostic biomarkers.

## Materials and methods

Detailed descriptions of databases and datasets (ONCOMINE, GEPIA, CCLE datasets, EMBL-EBL cell line data, LinkedOmics datasets) are provided in Supplementary Materials and methods.

### Breast cancer patients’ samples and ethics statement

This study was reviewed and approved by the ethical committee of the King Faisal Specialist Hospital and Research Centre (KFSH&RC, RAC # 2210002). Written informed consent was obtained from all patients according to institutional guidelines. Breast cancer patients with invasive ductal carcinoma had their excised tumor samples collected both before and after chemotherapy. Patients had at least three cycles of neoadjuvant (NAC) chemotherapy with either docetaxel (T) and doxorubicin (A) or epirubicin (E) and cyclophosphamide (C). All of the participants had an excisional biopsy with a needle before NAC. Following treatment, residual tumors were surgically removed. Patients were treated according to the neoadjuvant chemotherapy 2018 NCCN standard BC guidelines. The patient’s response to treatment was assessed according to RECIST 1.1 criteria. Accordingly, complete response (CR) was classified as the treatment response group. Conversely, patients with stable (SD) or progressing (PD) disease were classified as treatment-resistant or non-responsive.

### Cell culture

We used four BC cell lines: MCF-7, MB-231, BT-20, and SKBR3. All BC cell lines were purchased from ATCC (American Type Culture Collection, USA), and they were cultured using standard cell culture protocol in RPMI1640 medium (GIBCO, USA) supplemented with 10% FBS and penicillin and streptomycin cocktail. Each cell line was confirmed by short tandem repeat profiling using the GenePrint 10 system (Promega, USA). Cell cultures were tested routinely for the presence of *mycoplasma* using the MycoAlert mycoplasma detection kit (Lonza, USA).

### Cell viability assay

Cells were grown in 96-well plates at a density of 2000 cells per well. For cell proliferation assays, cell counts were assessed every 24 hours for 3 days following siRNA administration. Cell viability was evaluated 72 hours following treatment with cisplatin in escalating doses. WST-1 Viability Reagent was used to assess cell viability (Roche, USA). The amount of light detected by a microplate reader was used to calculate the number of live cells (Bio-Rad, USA). At least three different tests were run.

### Annexin V flow cytometry (apoptosis assay)

BT-20, MCF-7, MDA-MB-231, and SKRB3 cells were grown in standard culture conditions and treated with si-control and si-lncRNA DLEU2. After treatment, cells were trypsinized, washed with PBS, added 5 μl of Annexin V-FITC (Invitrogen, USA), 5 μl of propidium iodide (PI), and incubated for 15 min at room temperature (25 °C). After that, 200 μl of 1× binding buffer was added, and run by flow cytometer (NovoCyte, USA), and analyzed the data by NovoExpress software. Analysis of stained cells was distinguished into four groups. Annexin V−/PI− as viable cells, annexin V+/PI− as early apoptotic cells, annexin V+P+ as late apoptotic, and V−PI+ as necrotic cells.

### LncRNA-DLEU2 and ROR1 siRNA transfection

Cell transfection was carried out with Lipofectamine RNAi Max (ThermoFisher Scientific, USA) following the manufacturer’s standard methods. LncRNA DLEU2 siRNA, which targets lncRNA DLEU2 transcription (GCTTACACTTATGGAGCTA), and a negative control si-control (GCTCACATTGGTGATACTA) were purchased from Add Gene (USA). Silencing ROR1 was performed using si-ROR1 (# AM16708; ThermoFisher Scientific, USA) or scrambled siRNA (si-control).

### Immunohistochemistry analysis

For immunostaining, primary tumor tissues excised from the patient were fixed in formalin and processed as previously described [15]. The ROR1 (Invitrogen, USA) expression levels were scored on four scales as described [13]. A score of ‘0’ was assigned if the cells in the tumor section had no positive staining with the ROR1 antibody; a score of ‘1’ was assigned for low-level expression of ROR1which is less than 50% (low expression of ROR1) of tumor cells expressed ROR1; a score of 2 was assigned if the tumor cells are positive for ROR1 staining on more than 50% (moderate-level staining of ROR1); a score of 3 was assigned if the ROR1 staining of tumors cells on above 50% of cells (highly positive staining). Two independent certified pathologists reviewed all samples stained with ROR1.

### Invasion and migration assay

Invasion and migration assays were performed as described previously [40]. Briefly, cells were treated with si-lncRNA-DLEU2 and si-ROR1 for 24 h in a Corning BioCoat Matrigel invasion chamber, or a migration assay was performed in a 24-well plate (8-μm pore size). The cell concentration was kept limited to 20,000 cells/well. Invaded or migrated cells were stained with 1% crystal violet dye. The number of positively stained cells was counted and photographed using a phase contrast microscope. Three randomly selected fields were counted and data were analyzed and presented as a bar graph.

### RNA extraction and qRT-PCR

Total RNA from BC patients’ tumors and cell lines was extracted using the Total Nucleic Acid Isolation kit for FFPE (ThermoFisher Scientific, USA) and the RNeasy Plus Mini kit (Qiagen, USA) according to the manufacturer’s standard protocols. The complementary RNA was reverse transcribed using the cDNA Reverse Transcription Kit (ThermoFisher Scientific, USA). The results were calculated using the 2-delta-deltaCt methods and standardized with GAPDH. The sequences of all primers are listed in Supplementary Table S3.

### Western blotting

Cells were lysed in RIPA buffer supplemented with a protease inhibitor cocktail and proteins were separated and transferred to PVDF membranes. The primary antibodies were added to the PVDF membrane with 5% normal fat dry milk: ROR1(cat# PA5-14726, Invitrogen, USA), E-cadherin (cat# sc-71008; Santa Cruz, USA), N-cadherin (cat# sc-59987; Santa Cruz, USA), Vimentin (cat# 5741; Cell Signaling, USA), TWIST1 (cat# 69366; Cell Signaling, USA), SNAIL1(cat# sc-271977; Santa Cruz, USA), BMI1 (cat# 5856; Cell Signaling, USA) and OCT3/4 (cat# sc-5279, Santa Cruz, USA). HRP conjugated goat anti-rabbit (cat# 31460, Invitrogen, USA), anti-mouse (cat# 62-6520; Invitrogen, USA), and anti-goat (cat# A27014; Invitrogen, USA) were used as secondary antibodies and visualized using an enhanced chemiluminescence imaging system. The protein signal intensity was determined and quantified by ImageJ (http://imagej.net) as described previously [40].

### Sphere formation assay

Sphere formation assay was performed as described previously [41]. Briefly, Cells were seeded in ultra-low attachment 6-well plates, and cell density was kept at 5000 cells/well. The culture medium consisted of DMEM/F12 (1:1, Gibco, USA) medium supplemented with 0.4% BSA (Sigma, USA), 1% penicillin and streptomycin, B27, 20 ng/ml hEGF (Sigma, USA), 5 μg/mL insulin (Sigma, USA), 20 ng/mL FGF (Sigma, USA), 50 ng/mL hydrocortisone (Sigma, USA) and 4μg/mL heparin (Sigma, USA). Sphere sizes greater than 50μm were counted and quantified using an inverted microscope (Olympus, Japan).

### Computational analysis TCGA RNA-sequence BC dataset

We retrieved the BC RNA-Seq data from The Cancer Genomics Atlas (TCGA) data portal (https://wwwcancer.gov/) from 1084 breast cancer patients from cBioPortal (https://www.cbioportal.org/study/summary?id=brca_tcga_2018). Raw count data and pre-filtered clinical annotated data corresponding to the respective samples were downloaded using an R package TCGAbiolinks (v.2.22.1) [42]. For further analysis, gene-level counts were log2-transformed normalized counts as RESM values. The lncRNA DLEU2-high and ROR1-high groups were defined as tumors with a lncRNA DLEU2 or ROR1 expression value higher than the median for all samples. Other samples with lncRNA DLEU2 or ROR1 expression value below the threshold (median) are defined as the low expression for respective markers.

### PubMed GEO microarray datasets

We obtained breast cancer microarray data from the PubMed Gene Expression Omnibus database under accession no. GSE87455 and GSE1456. The lncRNA DLEU2 and ROR1-high groups were defined as tumors with a lncRNA DLEU2 or ROR1 expression value higher than the median for all samples. Other samples with lncRNA DLEU2 or ROR1 expression value below the threshold (median) are defined as low expression of respective markers.

### Gene set enrichment analysis (GSEA)

We analyzed GSEA [43] on the primary microarray dataset GSE84755 [3]. One hundred twenty-two BC samples were ranked by their relative expression of ROR1 using the signal-to-noise ratio ranking metric. We emphasized GSEA on 3 major gene targets: BMI1, Hippo-YAP, and EMT targets. Each gene set was considered significant when the false discovery rate (FDR) was lower than 25% [43]. For each gene set tested, we determined fowling parameters; the gene set size (SIZE), the enrichment score (ES), the normalized ES (NES), the nominal *p*-value (NOM p-value), and the FDR q-value (FDR q-value). The FDR *q*-value was adapted for gene set size and for multiple hypothesis testing.

### Statistical analysis

All in vitro experiments were done in triplicates. The comparisons between the two groups were performed using the Student’s *t-test* and Wilcoxon test as indicated in the individual figures. The GEO dataset analysis for lncRNA DLUE2/ROR1-high and -low groups were adjusted by FDR using the Benjamini-Hochberg method. Overall disease-free survival was analyzed using the Kaplan–Meier survival curve and log-rank test. *P* value less than < 0.05 was considered significant. All statistical analyses were performed using R-Statistical software (version 4.2.0; 2022; https://cran.r-project.org/bin/macosx/).

### Supplementary information

Supplementary Figures and methods

Supplementary Table S1

Supplementary Table S3

Supplementary Table S2

Raw western blots

## Data Availability

All public data used and analyzed are freely available in respective databases.
