# Supplementary material for: Long noncoding RNA DLEU2 and ROR1 pathway induces epithelial-to-mesenchymal transition and cancer stem cells in breast cancer
Source: Cell Death Discov. 2024 Jan 31;10:61. doi: 10.1038/s41420-024-01829-3 (PMC10830457; doi:10.1038/s41420-024-01829-3)
Supplement: Supplementary file 1 — Supplementary Figures and methods [file 41420_2024_1829_MOESM1_ESM.pdf]

Supplementary information

Title: Long noncoding RNA DLEU2 and ROR1 pathway induces epithelial-to-mesenchymal transition and cancer stem cells in breast cancer

Islam et al.

Number of supplementary figures: 6 (six)

Supplementary Materials and Methods

ONCOMINE database analysis

ONCOMINE (https://www.oncomine.org), an online cancer microarray database was used to retrieve and analyze the levels of selective clinically significant lncRNAs in major human cancers as well as BC. We have used threshold levels to restrict our analysis as follows: p-value=0.01, fold change parameter 1.5, gene rank 15%, and data type was lncRNA expression level. In each case, the lncRNA expression of tumor and normal expression were compared for the clinical cancer samples. Type of cancer, fold change, Student's *t*-test, *P*-values, and sample size were abstracted for comparisons and statistical significance.

GEPIA datasets

GEPIA (Gene Expression Profiling Interactive Analysis) is a newly developed interactive web server that allows users to analyze RNA sequence expression data from tumor and normal samples for all cancer types. It combinedly uses data from The Cancer Genomic Atlas (TCGA) and Genotype Tissue Expression (GTEx) projects using a standard data processing pipeline (http://gepia.cancer-pku.cn). GEPIA allows a range of easy-to-use and customizable features including differential expression analysis of tumor and matched normal tissues for each type of cancer and pathological stages, survival and progression-free survival analysis, principal

component analysis, similar gene detection, correlation analysis, and dimensionality analysis [1].

CCLE dataset

The CCLE (Cancer Cell Line Encyclopedia) is a collaboration between the Broad Institute and Novartis Institutes for Biomedical Research and its Genomic Institute of the Novartis Research Foundation to conduct a detailed genetic and pharmacologic characterization of a large panel of human cancer models. It developed an integrated computational analysis that links distinct pharmacologic vulnerabilities to genomics patterns and translates cell line integrative genomics into cancer patient stratification [2]. The CCLE platform provides public access to genomic data, analysis, and visualization for over 1100 cell lines from all types of cancer. The lncRNA-DLEU2 expression in BC cell lines is verified by the CCLE dataset.

EMBL-EBI cell line data

European Bioinformatic Institute (EMBL-EBI) is an international government organization which provides free and open access to a range of bioinformatics applications for sequence analysis [3]. We have verified lncRNA DLEU2 expression from CCLE cell line data by the EMBL-EBI cell line dataset.

LinkedOmics dataset

LinkedOmics (http://www.linkedomics.orglogin.php) is a newly developed and unique tool in the software ecosystem for disseminating data from large-scale cancer omics projects. It uses preprocessed and normalized data from the Broad-TCGA Firehose and Clinical Proteomic Tumor Analysis (CPTAC) data portal to reduce redundant efforts, and focus on the discovery

and interpretation of attributes association and thus complements existing cancer data portals [4].

References

1. Tang, Z., Li, C., Kang, B., Gao, G., Li, C., and Zhang, Z. (2017). GEPIA: A web server for cancer and normal gene expression profiling and interactive analyses. Nucleic Acids Res.
2. Barretina, J., Caponigro, G., Stransky, N., Venkatesan, K., Margolin, A.A., Kim, S., Wilson, C.J., Lehár, J., Kryukov, G. V., Sonkin, D., et al. (2012). The Cancer Cell Line Encyclopedia enables predictive modelling of anticancer drug sensitivity. Nature.
3. Madeira, F., Park, Y.M., Lee, J., Buso, N., Gur, T., Madhusoodanan, N., Basutkar, P., Tivey, A.R.N., Potter, S.C., Finn, R.D., et al. (2019). The EMBL-EBI search and sequence analysis tools APIs in 2019. Nucleic Acids Res.
4. Vasaikar, S. V., Straub, P., Wang, J., and Zhang, B. (2018). LinkedOmics: Analyzing multi-omics data within and across 32 cancer types. Nucleic Acids Res.

Supplementary Figures

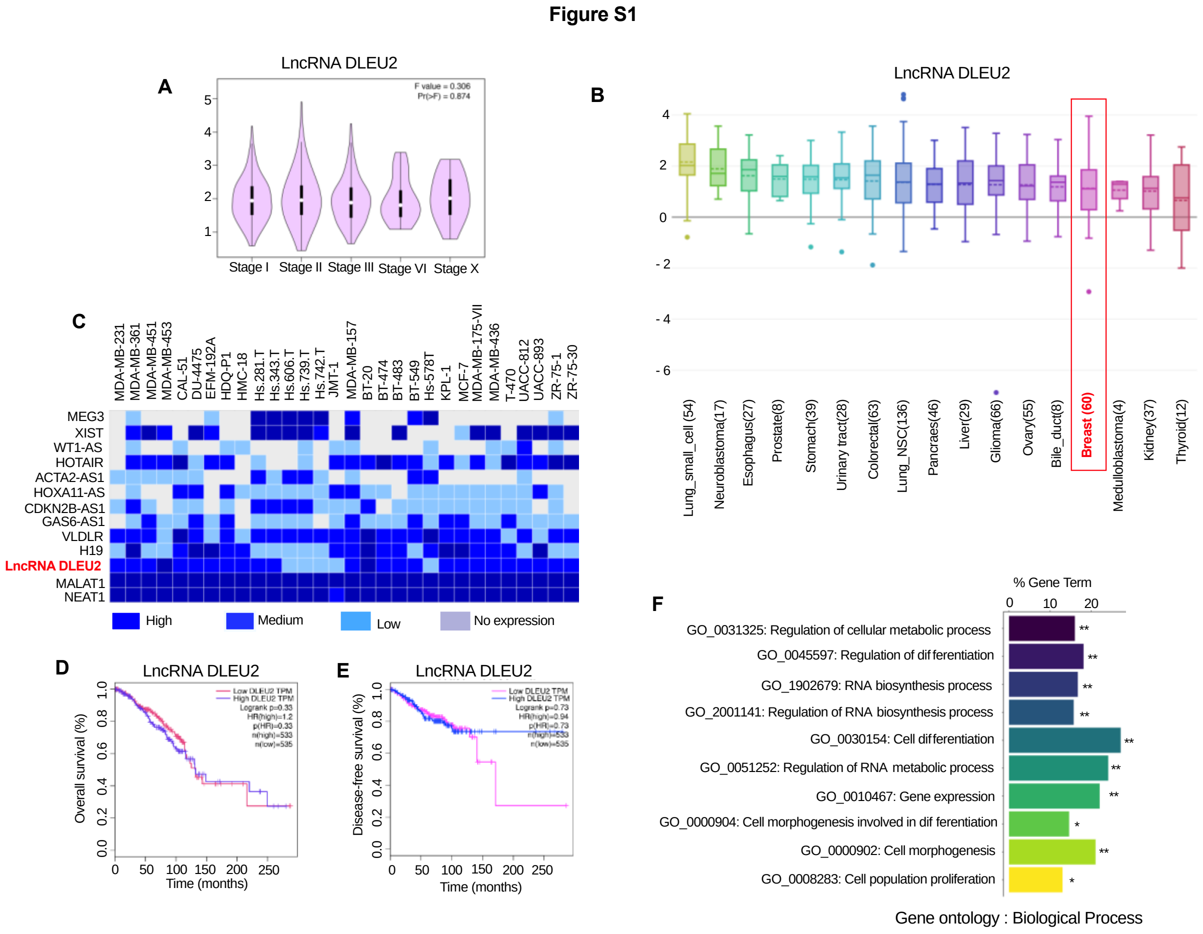

Figure S1: The expression lncRNA-DLEU2 in breast cancer

A Correlation between lncRNA DLEU2 expression and tumor stages in breast cancer patients (Data analyzed from GEPIA data set). **B** The expression of lncRNA DLEU2 in different cancer cell lines. Cell line information was retrieved from CCLE (breast cancer is highlighted in a red box). **C** The heatmap showing the expression of lncRNA DLEU2 in breast cancer cell lines analyzed by EMBL-EBI. **D-E** Prognostic value of mRNA level of lncRNA DLEU2 in breast cancer patients analyzed from GEPIA data set. **F** The functions of lncRNA DLEU2

and genes significantly related to lncRNA DLEU2 changes were predicted by the analysis of gene ontology (GO) by DAVID (Database for Annotations, Visualization, and Integrated Discovery) tools (https://david.ncifcrf.gov/summary.jsp). GO analysis predicted the functional roles of target host genes based on Biological Process (BP).

Figure S2

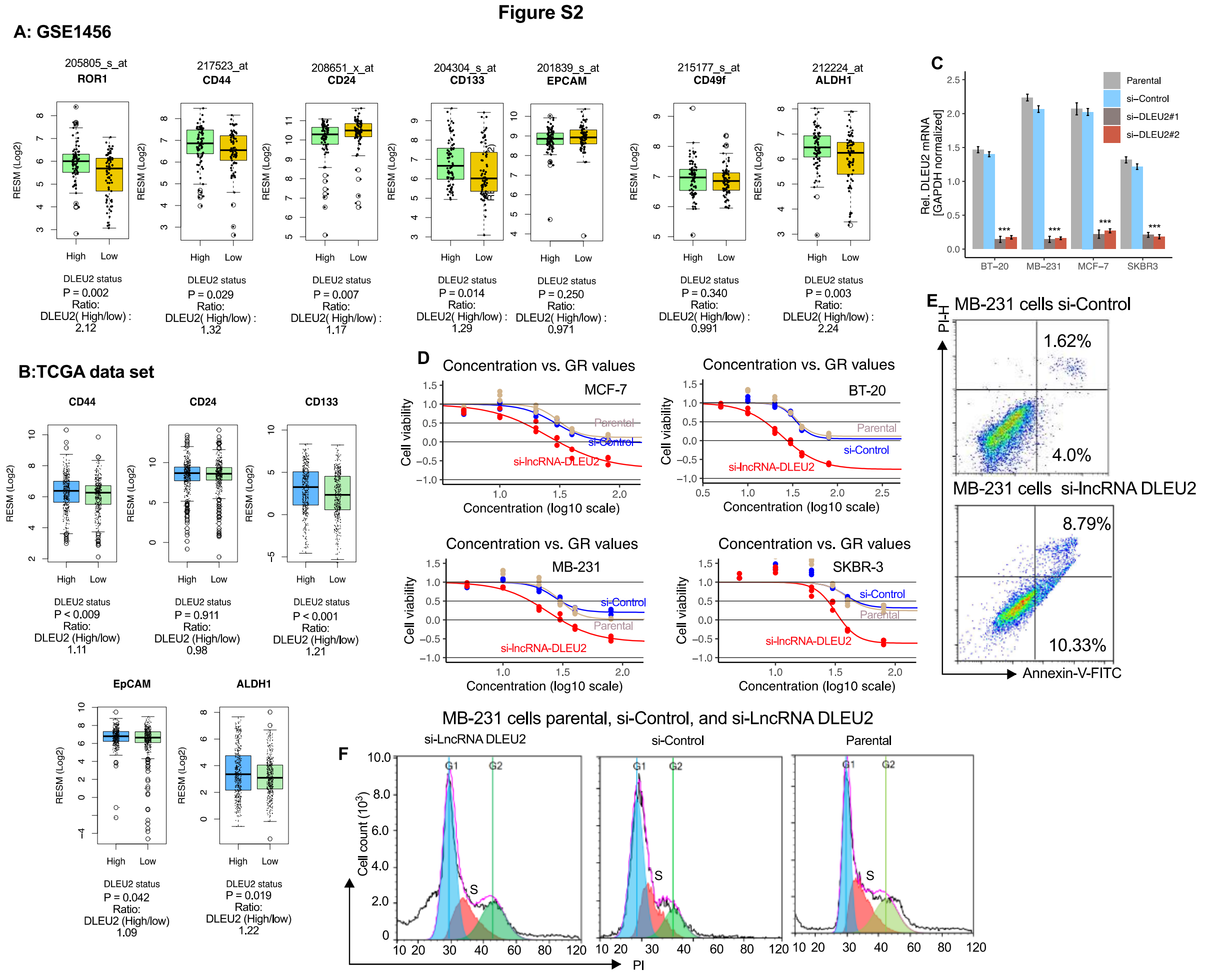

Figure S2: Regulation of cancer stem cells by lncRNA DLEU2

A Comparison of cancer stem cells (CSC)-related genes between lncRNA DLEU2-high and -low groups using the PubMed Gene Expression Omnibus (GEO) dataset (accession no. GSE1456). The boxes show the interquartile range (25th to 75th), and the horizontal lines inside the boxes indicate the median. Whiskers indicate the minimum and maximum values. The ratio was calculated by dividing the mRNA expression of the high group by the

expression of the low group. The *P* values were calculated using the Wilcoxon signed rank test. **B** Comparisons of cancer stem cells (CSC)-related genes between lncRNA DLEU2-high and -low groups using TCGA breast cancer dataset. The boxes represent the inter-quartile range (25th to 75th), and the horizontal lines inside the boxes indicate the median. Whiskers indicate the minimum and maximum values. The ratio is calculated by dividing the mRNA expression of the high and low groups. The *P* values were calculated using the Wilcoxon signed rank test. **C** LncRNA DLEU2 expression in parental, si-control, and si-lncRNA DLEU2 treated breast cancer cells. The expression differences between si-control and si-lncRNA-DLEU2 were compared. Student *t*-test was used to calculate the significance level. ***$P < 0.0001$. **D** Cell viability assay of breast cancer cells after cisplatin treatment. Cisplatin concentration ranges from 5 to 80 μM. The viability of cells was assessed for 72 hours of cisplatin treatment. **E** Flow cytometry analysis of cellular apoptosis measured after cells exposed to si-control and si-lncRNA DLEU2.

Figure S3

A; PubMed GEO dataset (GSE1456) dataset EMT gene analysis

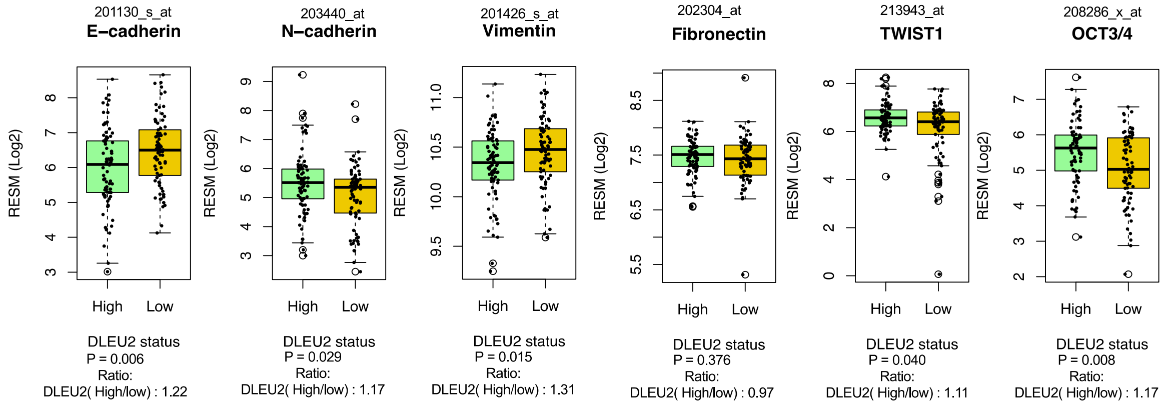

B: TCGA breast dataset EMT gene analysis

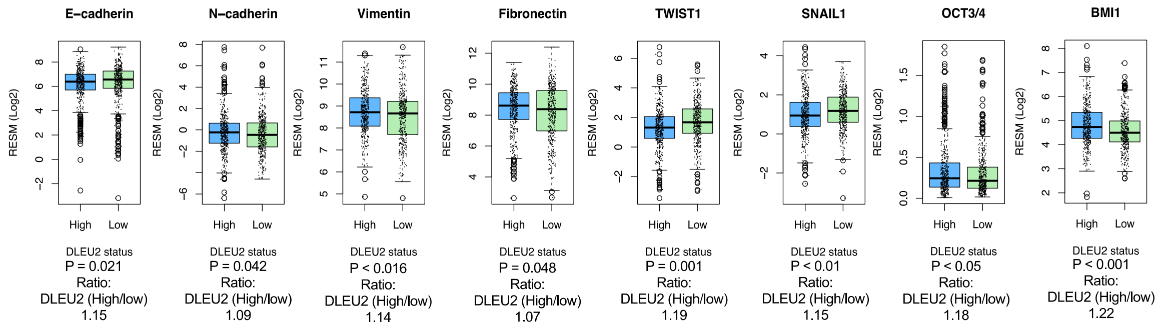

Figure S3: Comparisons of EMT-related genes between lncRNA DLEU2-high and -low groups from GEO database accession no. GSE1456.

A Comparison of epithelial-mesenchymal (EMT)- related genes between lncRNA DLEU2-high and -low groups using GEO database accession no. GSE1456. The boxes represent the inter-quartile range (25th to 75th), and the horizontal lines inside the boxes indicate the median. Whiskers indicate the minimum and maximum values. The lncRNA DLEU2 1-high group was defined as tumors with a lncRNA DLEU2 expression value higher than the median for all samples. The ratio is calculated by dividing the mRNA expression of the high group by the expression of the low group. The *P* values were calculated using the Wilcoxon signed rank test. **B** Comparisons of epithelial-mesenchymal (EMT)- related genes between

lncRNA DLEU2-high and -low groups using TCGA breast cancer dataset. The boxes represent the inter-quartile range (25th to 75th), and the horizontal lines inside the boxes indicate the median. Whiskers indicate the minimum and maximum values. The ratio is calculated by dividing the mRNA expression of the high group by the expression of the low group. The *P* values were calculated using the Wilcoxon signed rank test.

Figure S4

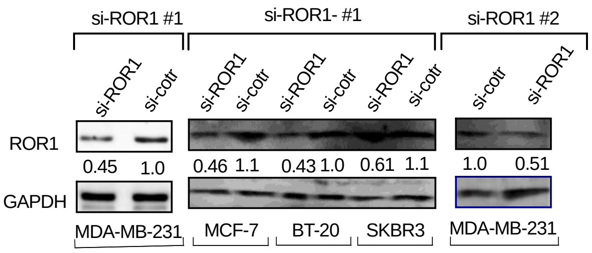

Supplementary Figure S4: Western blot analysis of ROR1 in two different siRNA-ROR1 in MB-231, MCF-7, BT-20, and SKBR3 breast cancer cell lines.

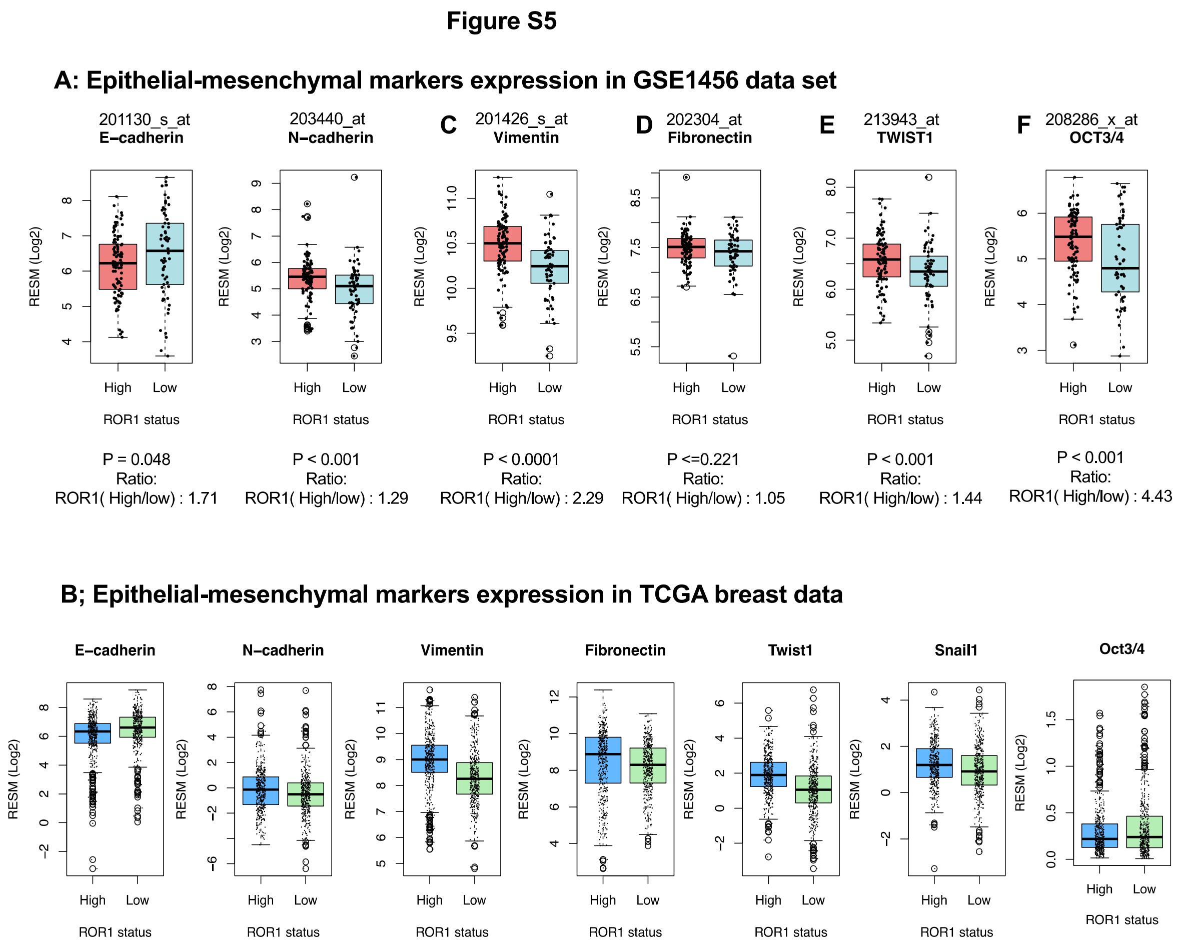

Figure S5: Comparisons of CSC-related genes between ROR1-high and -low groups from GEO database accession no. GSE 1456.

A, Comparisons of EMT-related genes between ROR1-high and -low groups using PubMed Gene Expression Omnibus (GEO) dataset (accession no. GSE1456)**.** The boxes represent the inter-quartile range (25th to 75th), and the horizontal lines inside the boxes indicate the median. Whiskers indicate the minimum and maximum values. ROR1 -high group was defined as tumors with a ROR1 expression value higher than the median for all samples. The ratio is calculated by dividing the mRNA expression of the high and low groups. *P* values were calculated using the Wilcoxon signed rank test.

B, Comparisons of EMT-related genes between ROR1-high and -low groups using TCGA

breast cancer dataset. The boxes represent the inter-quartile range (25th to 75th), and the horizontal lines inside the boxes indicate the median. Whiskers indicate the minimum and maximum values. The ratio is calculated by dividing the mRNA expression of the high low group. The *P* values were calculated using the Wilcoxon signed rank test.

Figure S6

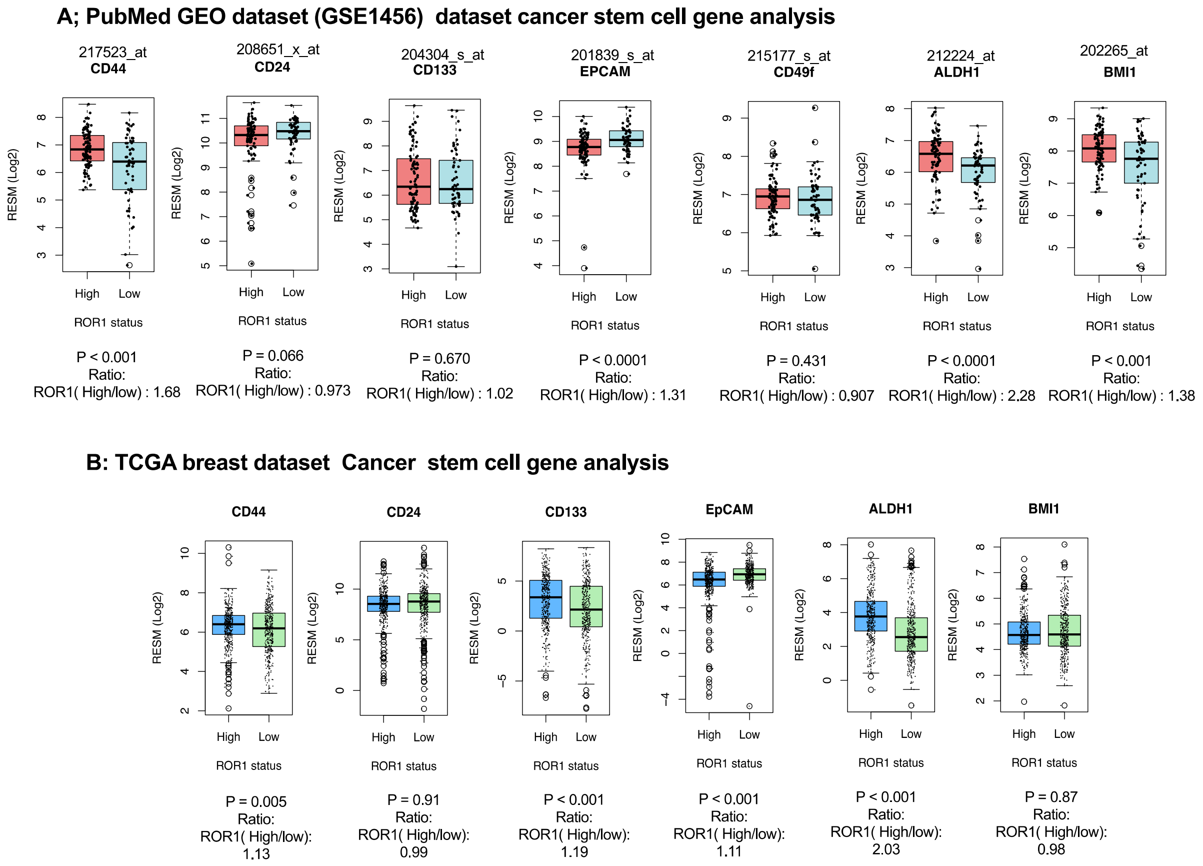

Figure S6: Comparison of breast cancer stem cell markers genes between ROR1-high and -low groups from GEO database accession no. GSE1456 and TCGA breast cancer data.

A, Comparisons of CSC-related genes between ROR1-high and -low groups using PubMed Gene Expression Omnibus (GEO) dataset (accession no. GSE1456)**.** The boxes represent the interquartile range (25th to 75th), and the horizontal lines inside the boxplot indicate the median. Whiskers in the top and bottom indicate the maximum and minimum values of the data. The ROR1-high group was defined as tumors with an ROR1 expression value higher than the median for all samples. The ratios were calculated by dividing the mRNA expression value of high group by the expression value of the low group. *P* values were

calculated using Wilcoxon signed rank test.

B, Comparisons of CSC-related genes between ROR1-high and -low groups using TCGA breast cancer dataset. The boxes represent the inter-quartile range (25th to 75th), and the horizontal lines inside the boxes indicate the median. Whiskers indicate the minimum and maximum values. The ratio is calculated by dividing the mRNA expression of high and low groups. The *P* values were calculated using the Wilcoxon signed rank test.
