# Supplementary material for: Long noncoding RNA DLEU2 and ROR1 pathway induces epithelial-to-mesenchymal transition and cancer stem cells in breast cancer
Source: Cell Death Discov. 2024 Jan 31;10:61. doi: 10.1038/s41420-024-01829-3 (PMC10830457; doi:10.1038/s41420-024-01829-3)
Supplement: Supplementary file 2 — Supplementary Table S1 [file 41420_2024_1829_MOESM2_ESM.docx]

| ***Supplementary Table S1. The significant changes of lncRNAs expression level between different types of breast tumors and normal breast tissues (Oncomine Dataset)*** | | | | | | | |
| --- | --- | --- | --- | --- | --- | --- | --- |
| **LncRNAs** | **Source of datasets /references** | **Reporters** | **Normal**  **(no of cases)** | **Tumors (No of cases)** | **Fold change** | **t-value** | **p-value** |
| HOTAIR | ^1^ | A_32_P168441 | Breast (61) | Invasive Breast Carcinoma (76) | 3.830 | 6.848 | 1.36E-10 |
|  | ^1^ | A_32_P168441 | Breast (61) | Invasive Ductal Breast Carcinoma (389) | 2.983 | 8.032 | 4.43E-13 |
|  | ^1^ | A_32_P168442 | Breast (61) | Invasive Lobular Breast Carcinoma (36) | 5.379 | 5.379 | 1.88E-11 |
|  | ^2^ | 239153_at | Breast (7) | Ductal Breast Carcinoma (40) | 1.670 | 1.750 | 0.051 |
|  | Wooster Cell Line Statistics |  |  | Invasive Breast Carcinoma | 2.898 | 4.979 | 3.62-E5 |
|  | Adi CellLine Statistics |  |  | Invasive Breast Carcinoma | 4.539 | 4.681 | 1.68-E5 |
|  | Barretina CellLine |  |  | Invasive Breast Carcinoma | 3.771 | 6.816 | 2.96E-9 |
| H19 | ^3^ | 224997_x_at | Breast (20) | Invasive Lobular Breast Carcinoma (5) | 2.176 | 2.827 | 0.008 |
| NEAT1 | ^3^ | 227062_at | Breast (20) | Invasive Lobular Breast Carcinoma (5) | 1.785 | 2.395 | 0.022 |
|  | ^4^ | IMAGE:510273 | Breast (3) | Invasive Ductal Breast Carcinoma (38) | 2.827 | 4.553 | 0.006 |
|  | ^4^ | MAGE:510273 | Breast (3) | Lobular Ductal Breast Carcinoma (21) | 2.291 | 3.742 | 0.013 |
| ANRIL | ^2^ | 1559884_at | Breast (7) | Ductal Breast Carcinoma (40) | 1.128 | 4.108 | 9.70E-5 |
| ACTA2 | ^2^ | 215787_at | Breast (7) | Ductal Breast Carcinoma (40) | 1.106 | 1.706 | 0.051 |
| DLEU2 | ^1^ | A_23_P22072 | Breast (61) | Invasive Ductal Breast Carcinoma (76) | 2.244 | 8.889 | 2.03E-15 |
|  | ^1^ | A_23_P22075 | Breast (61) | Invasive Ductal Breast Carcinoma (389) | 1.828 | 11.122 | 1.53E-20 |
|  | ^1^ | A_23_P22075 | Breast (61) | Invasive Lobular Breast Carcinoma (36) | 1.876 | 6.423 | 1.77E-8 |
|  | ^2^ | 216870_x_at | Breast (7) | Invasive Ductal Breast Carcinoma (40) | 1.792 | 5.358 | 4.08E-6 |
|  | ^3^ | 239936_at | Breast (20) | Invasive Ductal Breast Carcinoma (5) | 2.992 | 2.503 | 0.013 |
|  | ^3^ | 1556820_a_at | Breast (20) | Invasive Lobular Breast Carcinoma (5) | 2.722 | 2.368 | 0.025 |
|  | ^4^ | \|  \| \| --- \| \| IMAGE:270136 \| | Breast (3) | Invasive Ductal Breast Carcinoma (37) | 1.224 | 4.268 | 8.91E-4 |
|  | ^1^ | ILMN_1784046 | Breast (144) | Invasive Lobular Breast Carcinoma (148) | 1.022 | 2.095 | 0.019 |
|  | ^5^ | 114 | Breast (4) | Invasive Ductal Breast Carcinoma (154) | 1.199 | 1.978 | 0.057 |
| GAS6 | ^6^ | IMAGE:796181 | Breast (3) | Lobular Breast Carcinoma (4) | 1.205 | 2.747 | 0.038 |
|  | ^6^ | IMAGE:796181 | Breast (3) | Ductal Breast Carcinoma (36) | 1.213 | 2.777 | 0.032 |
|  | ^7^ | 13-113568657 | Breast (111) | Ductal Breast Carcinoma (5) | 1.040 | 2.463 | 0.035 |
| XIST | ^8^ | W02228 | Breast (4) | Invasive Lobular Breast Carcinoma (5) | 3.283 | 3.005 | 0.016 |
|  | ^8^ | W02228 | Breast (4) | Invasive Ductal Breast Carcinoma (26) | 2.886 | 2.203 | 0.037 |
| MALAT1 | ^8^ | AW176909 | Breast (9) | Invasive Lobular Breast Carcinoma (7) | 2.038 | 1.823 | 0.046 |
|  | ^8^ | BE086548 | Breast (7) | Invasive Ductal Breast Carcinoma (28) | 1.558 | 1.707 | 0.051 |
|  | ^4^ | IMAGE:253009 | Breast (3) | Invasive Ductal Breast Carcinoma (37) | 2.166 | 4.473 | 6.14E-4 |
|  | ^4^ | IMAGE:253009 | Breast (3) | Lobular Breast Carcinoma (21) | 1.778 | 2.651 | 0.009 |
|  | ^7^ | A_23_P21143 | Breast (61) | Invasive Lobular Breast Carcinoma (36) | 1.516 | 3.599 | 2.83E-4 |
|  | ^7^ | A_23_P21143 | Breast (61) | Invasive Ductal Breast Carcinoma (389) | 1.498 | 5.004 | 1.30E-6 |
|  | ^7^ | A_24_P873659 | Breast (61) | Invasive Breast Carcinoma (76) | 1.780 | 3.521 | 2.98E-4 |
|  | ^2^ | 223578_x_at | Breast (7) | Ductal Breast Carcinoma (40) | 1.870 | 4.123 | 8.45E-5 |
| MEG3 | ^3^ | 229557_at | Breast (20) | Invasive Lobular Breast Carcinoma (5) | 1.567 | 2.260 | 0.023 |
|  | ^1^ | AK055725_1_2644 | Breast (61) | Invasive Ductal/Lobular Breast Carcinoma (3) | 2.405 | 2.527 | 0.046 |
|  | ^1^ | AK055725_1_2644 | Breast (61) | Invasive Lobular Breast Carcinoma (36) | 1.843 | 3.470 | 3.92E-4 |
|  | ^1^ | AK055725_1_2644 | Breast (61) | Invasive Ductal Breast Carcinoma (389) | 1.652 | 3.528 | 3.72E-4 |
|  | ^1^ | AK055725_1_2644 | Breast (61) | Invasive Breast Carcinoma (76) | 1.496 | 2.376 | 0.010 |
| VLDLR | No relevant significance samples found across the breast cancer vs normal tissues | | | | | | |
| WT1 | ^5^ | 32664 | Breast (4) | Invasive Breast Carcinoma (154) | 5.024 | 8.427 | 7.58E-5 |
|  | ^1^ | NM_024426_2_2917 | Breast (61) | Invasive Breast Carcinoma (76) | 8.120 | 9.009 | 8.85E-16 |
|  | ^1^ | A_23_P116272 | Breast (61) | Invasive Ductal Breast Carcinoma (389) | 2.971 | 9.332 | 2.90E-16 |
|  | ^1^ | A_23_P116272 | Breast (61) | Invasive Lobular Breast Carcinoma (36) | 2.590 | 4.920 | 3.69E-6 |
|  | ^2^ | 206067_s_at | Breast (7) | Ductal Breast Carcinoma (40) | 1.874 | 4.338 | 5.05E-5 |
|  | ^1^ | ILMN_1802174 | Breast (144) | Invasive Ductal Breast Carcinoma (1556) | 1.211 | 14.521 | 2.31E-38 |
|  | ^1^ | ILMN_1802174 | Breast (144) | Invasive Ductal/Lobular Breast Carcinoma (90) | 1.097 | 5.193 | 2.85E-7 |
|  | ^1^ | ILMN_1802174 | Breast (144) | Invasive Breast Carcinoma (21) | 1.369 | 2.485 | 0.011 |
|  | ^1^ | ILMN_1802174 | Breast (144) | Invasive Lobular Breast Carcinoma (148) | 1.107 | 5.053 | 4.31E-7 |
|  | ^1^ | ILMN_1802174 | Breast (144) | Breast Carcinoma (14) | 1.134 | 2.320 | 0.018 |
|  | ^4^ | IMAGE:503338 | Breast (3) | Invasive Ductal Breast Carcinoma (38) | 2.033 | 2.239 | 0.059 |
| LNP1 | ^7^ | 03-101630293 | Breast (111) | Medullary breast carcinoma (4) | 1.112 | 3.741 | 0.017 |

**References**

1 Curtis C, Shah SP, Chin SF, Turashvili G, Rueda OM, Dunning MJ *et al.* The genomic and transcriptomic architecture of 2,000 breast tumours reveals novel subgroups. *Nature* 2012. doi:10.1038/nature10983.

2 Richardson AL, Wang ZC, De Nicolo A, Lu X, Brown M, Miron A *et al.* X chromosomal abnormalities in basal-like human breast cancer. *Cancer Cell* 2006. doi:10.1016/j.ccr.2006.01.013.

3 Turashvili G, Bouchal J, Baumforth K, Wei W, Dziechciarkova M, Ehrmann J *et al.* Novel markers for differentiation of lobular and ductal invasive breast carcinomas by laser microdissection and microarray analysis. *BMC Cancer* 2007. doi:10.1186/1471-2407-7-55.

4 Zhao H, Langerød A, Ji Y, Nowels KW, Nesland JM, Tibshirani R *et al.* Different gene expression patterns in invasive lobular and ductal carcinomas of the breast. *Molecular Biology of the Cell* 2004. doi:10.1091/mbc.E03-11-0786.

5 Glück S, Ross JS, Royce M, McKenna EF, Perou CM, Avisar E *et al.* TP53 genomics predict higher clinical and pathologic tumor response in operable early-stage breast cancer treated with docetaxel-capecitabine ± Trastuzumab. *Breast Cancer Research and Treatment* 2012. doi:10.1007/s10549-011-1412-7.

6 Perou CM, Sørile T, Eisen MB, Van De Rijn M, Jeffrey SS, Ress CA *et al.* Molecular portraits of human breast tumours. *Nature* 2000. doi:10.1038/35021093.

7 Zwiener I, Frisch B, Binder H, Goodspeed A, Heiser LM, Gray JW *et al.* The genomic and transcriptomic architecture of 2,000 breast tumours reveals novel subgroups. *Nature* 2017.

8 Radvanyi L, Singh-Sandhu D, Gallichan S, Lovitt C, Pedyczak A, Mallo G *et al.* The gene associated with trichorhinophalangeal syndrome in humans is overexpressed in breast cancer. *Proceedings of the National Academy of Sciences of the United States of America* 2005. doi:10.1073/pnas.0500904102.
