# Supplementary material for: Long noncoding RNA DLEU2 and ROR1 pathway induces epithelial-to-mesenchymal transition and cancer stem cells in breast cancer
Source: Cell Death Discov. 2024 Jan 31;10:61. doi: 10.1038/s41420-024-01829-3 (PMC10830457; doi:10.1038/s41420-024-01829-3)
Supplement: Supplementary file 3 — Supplementary Table S3 [file 41420_2024_1829_MOESM3_ESM.docx]

| Supplementary Table S3: List of primers | | |
| --- | --- | --- |
| Genes name | Forward primer | Reverse primer |
| BMI1 | CCAGGGCTTTTCAAAAATGA | CCGATCCAATCTGTTCTGGT |
| OCT3/4 | GTACTCCTCGGTCCCTTTCC | TCTGCTGGAGGCTGAGGTAT |
| LncRNA-DLEU2 | GCGGGTACTTATCTCCGACC | GTTTCCCAGTCGCTGTTCGT |
| ROR1 | CAACAAGAAGCCTCCCTAATGG | CCTGAGTGACGGCACCTAGAA |
| CD44 | TGA ATA TAA CCT GCC GCT TTG | GTC ATA CTG GGA GGT GTT GGA |
| CD24 | CTG CCT CGA CAC ACA TAA ACC TT | CAT CTA AGC ATC AGT GTG TGA CCA |
| CD133 | AAGCATTGGCATCTTCTATGG | AAGCACAGAGGGTCATTGAGA |
| EpCAM | GAA TGG CAA AGT ATG AGA AGG CTG A | TCC CAC GCA CAC ACA TTT GTA A |
| ALDH1 | CGGGAAAAGCAATCTGAAGAGGG | GATGCGGCTATACAACACTGGC |
| CD49f | ACC CAG ATA TTG CAG TTG GA | TTC GAT CAA GGT CCA TGT TT |
| E-cadherin | GCCTCCTGAAAAGAGAGTGGAAG | TGGCAGTGTCTCTCCAAATCCG |
| N-cadherin | GGCATACACCATG CCATCTT | GTGCATGAAGGACAGCCTCT |
| Vimentin | AGGCAAAGCAGGAGTCCACTGA | ATCTGGCGTTCCAGGGACTCAT |
| Fibronectin | ACAACACCGAGGTGACTGAGAC | GGACACAACGATGCTTCCTGAG |
| TWIST1 | GGAGTCCGCAGTCTTACGAG | TCTGGAGGACCTGGTAGAGG |
| SNAIL1 | GAAAGGCCTTCAACTGCAAA | TGACATCTGAGTGGGTCTGG |
| GAPDH | CATCACCATCTTCCAGGAGC | ATGCCAGTGAGCTTCCCGTC |
