# Supplementary material for: Long noncoding RNA DLEU2 and ROR1 pathway induces epithelial-to-mesenchymal transition and cancer stem cells in breast cancer
Source: Cell Death Discov. 2024 Jan 31;10:61. doi: 10.1038/s41420-024-01829-3 (PMC10830457; doi:10.1038/s41420-024-01829-3)
Supplement: Supplementary file 4 — Supplementary Table S2 [file 41420_2024_1829_MOESM4_ESM.docx]

| **Supplementary Table S2: Pathological and clinical information of thirty-eight breast cancer patients’ tumors who received Neoadjuvant chemotherapies** | | | | |
| --- | --- | --- | --- | --- |
| Patients SL. No. | Breast cancer Subtype Status | Treatment | ROR1 expression Score  Pre-treatment Post-treatment | |
| 1 | ER^-^/PR^-^/HER2^+^ | TE | 2 | 3 |
| 2 | ER^+^/PR^-^/HER^-^ | TEC | 1 | 3 |
| 3 | ER^+^/PR^-^/HER^-^ | TEC | 2 | 2 |
| 4 | ER^-^/PR^-^/HER2^+^ | TEC | 2 | 3 |
| 5 | ER^-^/PR^-^/HER^-^ | TE | 2 | 3 |
| 6 | ER^+^/PR^+^/HER2^-^ | TEC | 3 | 3 |
| 7 | ER^-^/PR^-^/HER2^+^ | TE | 1 | 1 |
| 8 | ER^-^/PR^-^/HER2^+^ | TE | 2 | 3 |
| 9 | Undefined type | TEC | 1 | 2 |
| 10 | ER^-^/PR^-^/HER^-^ | TE | 2 | 2 |
| 11 | ER^+^/PR^-^/HER^-^ | TEC | 1 | 3 |
| 12 | ER^-^/PR^-^/HER2^+^ | TEC | 2 | 3 |
| 13 | ER^-^/PR^-^/HER^-^ | TE | 1 | 3 |
| 14 | ER^-^/PR^-^/HER2^+^ | TEC | 3 | 3 |
| 15 | ER^-^/PR^-^/HER^-^ | TE | 1 | 2 |
| 16 | ER^-^/PR^-^/HER^-^ | TE | 1 | 1 |
| 17 | ER^-^/PR^-^/HER2^+^ | TEC | 1 | 3 |
| 18 | ER^+^/PR^+^/HER2^-^ | TEC | 2 | 2 |
| 19 | ER^+^/PR^-^/HER^-^ | TE | 2 | 0 |
| 20 | ER^-^/PR^-^/HER2^+^ | TE | 1 | 3 |
| 21 | ER^-^/PR^-^/HER2^+^ | TEC | 2 | 3 |
| 22 | ER^+^/PR^+^/HER2^-^ | EC | 2 | 3 |
| 23 | ER^+^/PR^-^/HER^-^ | TE | 1 | 1 |
| 24 | ER^-^/PR^-^/HER^-^ | TE | 1 | 3 |
| 25 | ER^+^/PR^-^/HER^-^ | TEC | 2 | 3 |
| 25 | ER^-^/PR^-^/HER2^+^ | TE | 2 | 3 |
| 27 | ER^-^/PR^-^/HER^-^ | TE | 2 | 2 |
| 28 | ER^-^/PR^-^/HER2^+^ | TE | 1 | 2 |
| 29 | ER^+^/PR^+^/HER2^-^ | TE | 1 | 1 |
| 30 | ER^+^/PR^-^/HER^-^ | TE | 2 | 3 |
| 31 | ER^+^/PR^+^/HER2^-^ | TEC | 2 | 0 |
| 32 | ER^-^/PR^-^/HER2^+^ | TEC | 1 | 2 |
| 33 | ER^-^/PR^-^/HER^-^ | TE | 2 | 2 |
| 34 | ER^-^/PR^+^/HER^-^ | TEC | 1 | 0 |
| 35 | ER^-^/PR^+^/HER^-^ | TEC | 3 | 3 |
| 36 | ER^-^/PR^-^/HER2^+^ | TEC | 3 | 0 |
| 37 | ER^-^/PR^+^/HER^-^ | TE | 2 | 2 |
| 38 | ER^-^/PR^-^/HER^-^ | TE | 2 | 2 |

*TEC: Taxotere-Epirubicin-Cyclophosphamide; TE: Paclitaxel Docetaxel/Cyclophosphamide; TAC: Taxotere-doxorubicin-Cyclophosphamide; EC: Epirubicin-Cyclophophamide*
