# Supplementary material for: Long noncoding RNA DLEU2 and ROR1 pathway induces epithelial-to-mesenchymal transition and cancer stem cells in breast cancer
Source: Cell Death Discov. 2024 Jan 31;10:61. doi: 10.1038/s41420-024-01829-3 (PMC10830457; doi:10.1038/s41420-024-01829-3)

Supplementay materials: Uncropped/Raw western blot images for Figures 3E, 4F, 8A,,8C, 8D

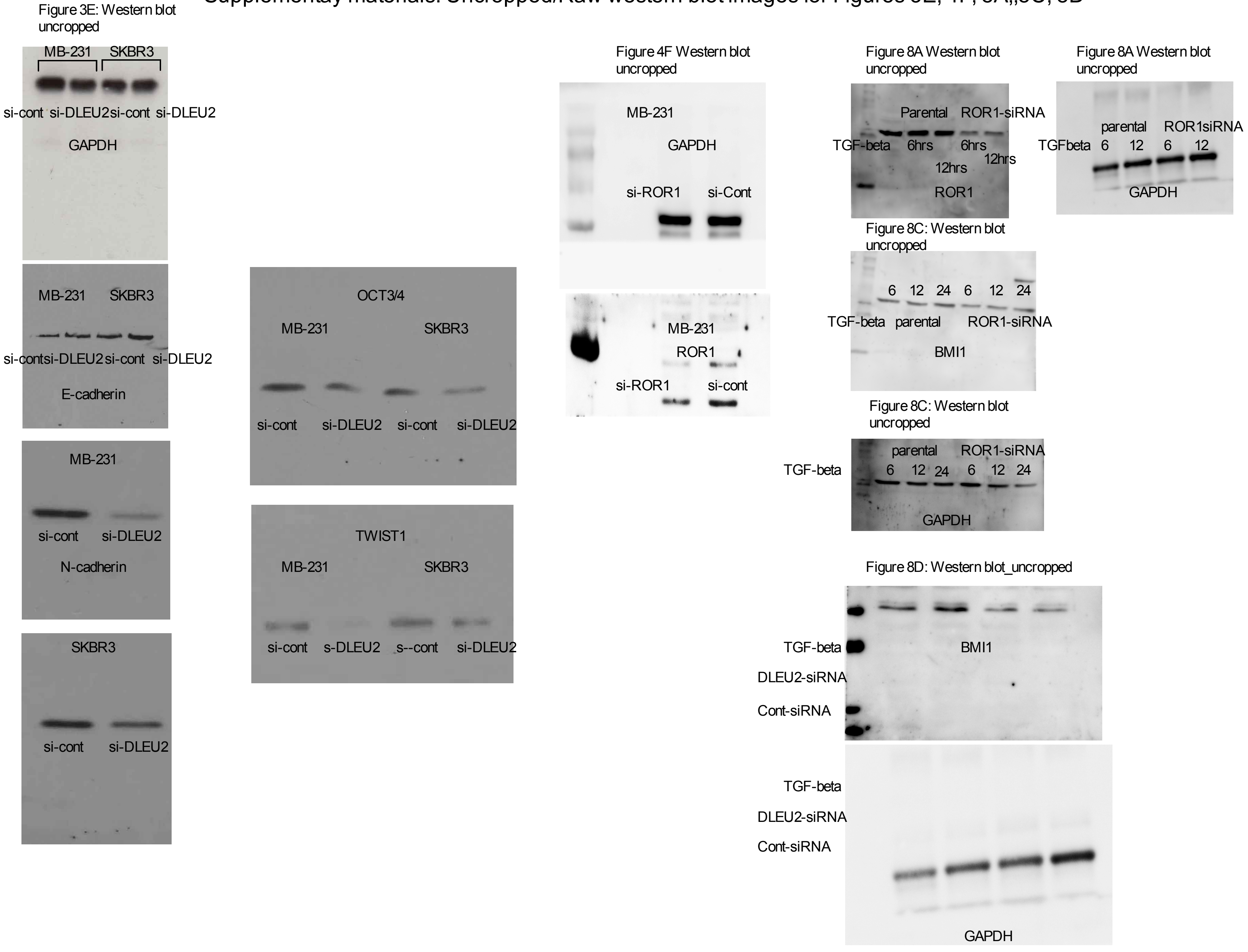

Supplement: Supplementary file 5 — Raw western blots [file 41420_2024_1829_MOESM5_ESM.pdf]
